# Applications in osteochondral organoids for osteoarthritis research: from pathomimetic modeling to tissue engineering repair

**DOI:** 10.3389/fbioe.2025.1629608

**Published:** 2025-07-23

**Authors:** Yingguang Jiao, Shanyu Lu, Jianwei Zhang, Junping Zhen

**Affiliations:** ^1^ College of Medical Imaging, Shanxi Medical University, Taiyuan, Shanxi, China; ^2^ Department of Imaging, Second Hospital of Shanxi Medical University, Taiyuan, Shanxi, China; ^3^ Molecular Imaging Laboratory, Second Hospital of Shanxi Medical University, Taiyuan, Shanxi, China

**Keywords:** osteoarthritis, osteochondral organoids, tissue engineering, drug screening, 3D bioprinting, pathomimetic modeling, hydrogels

## Abstract

Osteoarthritis (OA) is a prevalent degenerative joint disorder characterized by complex tissue interactions, featuring cartilage degradation, synovitis, and aberrant subchondral bone remodeling. Current therapies often fail to halt disease progression and typically lack comprehensive strategies targeting OA pathogenesis. Osteochondral organoids have recently emerged as innovative 3D biological models for investigating OA mechanisms and developing personalized therapies. These models recapitulate dynamic cell-cell and cell-matrix interactions within the articular microenvironment. This review evaluates progress in applying osteochondral organoids to osteoarthritis, focusing on their fabrication strategies, applications, and key challenges. It emphasizes their role in osteoarthritis modeling, drug screening, and cartilage regeneration, while exploring future directions for their development. Despite these advances, clinical translation of osteochondral organoids faces significant challenges, including standardization, vascularization, and immunomodulation. Future integration with organ-on-chip platforms, multi-omics, and AI promises to create more precise OA research models. Such integration will bridge the gap between bench research and clinical practice.

## 1 Introduction

Osteoarthritis (OA) is the most common degenerative joint disorder, whose prevalence correlates strongly with both population aging and rising obesity rates. According to statistics, approximately 35% of individuals over 60 years of age worldwide are affected by OA, which is primarily manifested by joint pain, dysfunction, and a significant decline in quality of life, causing a substantial burden on the socio-economy (Hunter et al., 2020; Yao Q. et al., 2023). OA pathogenesis extends beyond articular cartilage degeneration to include synovitis, subchondral bone remodeling abnormalities, and neuro-periosteal signaling dysregulation, reflecting complex multi-tissue and multi-pathway interactions (Yao Z. et al., 2023). These pathological imbalances severely restrict patients’ joint function and quality of life (Grässel et al., 2021).

Current OA treatments focus predominantly on symptom management, including oral NSAIDs, intra-articular corticosteroid/hyaluronic acid injections, and arthroplasty for advanced cases. However, these interventions often demonstrate limited durability, significant adverse effects, or high invasiveness (Yu and Hunter, 2015; Chen et al., 2021; Franchi et al., 2022). In recent years, a number of emerging technologies have garnered attention in the field of orthopedic research and practice. These include platelet-rich plasma (PRP) injections, disease-modifying anti-osteoarthritis drugs (DMOADs), and stem cell therapies (Jia et al., 2022; Zhang et al., 2024). Despite their therapeutic promise, these modalities have yet to achieve optimal clinical outcomes. Key challenges remain, particularly regarding osteochondral integration and sustained symptom alleviation.

Despite progress in OA research, current models fail to fully recapitulate the intricate 3D architecture and multi-tissue crosstalk of human joints. Traditional 2D models, while scalable, lack tissue architecture, extracellular matrix interactions, and biomechanical microenvironment, resulting in low physiological relevance and poor pharmacological predictability (Alnasser, 2025). Animal models, though widely used, suffer from interspecies differences (e.g., metabolism, immune response), leading to mechanistic misinterpretations (Zhang et al., 2022b). They are also costly, time-consuming, and prone to false positives/negatives in toxicity testing, contributing to low clinical translation rates (Huang et al., 2021). In contrast, osteochondral organoids replicate native joint cartilage with 3D structure, cellular heterogeneity, and functional properties, enabling more accurate modeling of *in vivo* microenvironments and cell-cell interactions (Yao et al., 2024). These organoids support high-throughput drug screening, with computational approaches further enhancing scalability (Boone et al., 2025). Additionally, patient-derived organoids improve individualized treatment and toxicity prediction. The advent of organoid technology has engendered a novel paradigm for osteoarthritis research. The induction of pluripotent stem cells (iPSCs) or mesenchymal stem cells (MSCs) in biomimetic scaffolds, in conjunction with specific growth factors and mechanical stimulation, facilitates the construction of microtissue models that exhibit the structural and functional characteristics of natural bone and cartilage (Shen et al., 2024). These osteochondral organoids are capable of mimicking the inflammatory response, matrix degradation, and nerve-bone signaling disorders associated with OA *in vitro* (Chen et al., 2022). Additionally, they serve as a versatile platform for drug screening and the evaluation of regenerative repair strategies. This review outlines the fabrication of osteochondral organoids using diverse stem cell sources, hydrogel scaffolds, advanced biomanufacturing, and directed differentiation techniques. It also evaluates their applications, current challenges, and future research directions.

## 2 Construction strategies for osteochondral organoids

**FIGURE 1:**
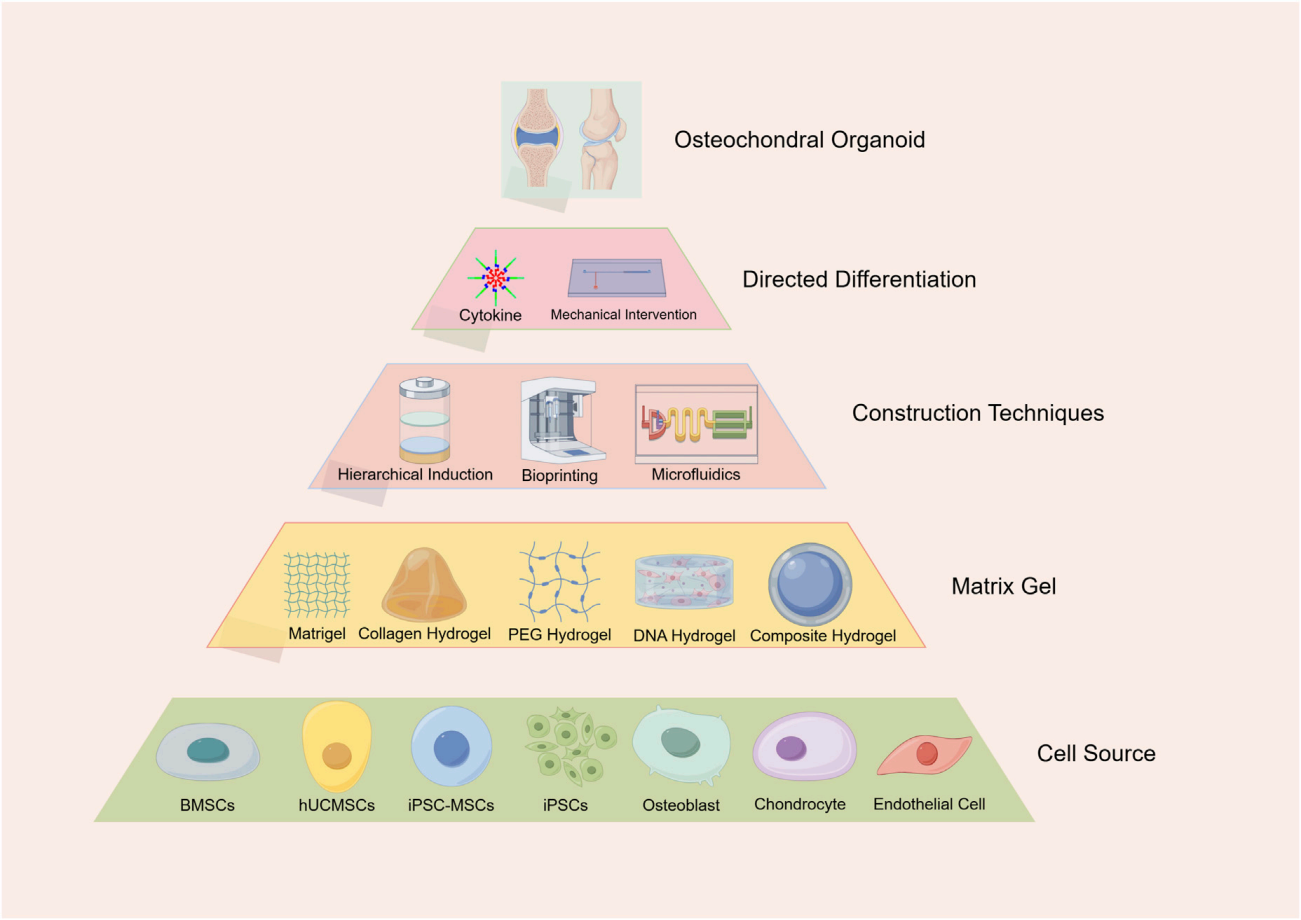
Strategies for the construction of osteochondral organoids. This pyramid outlines strategies for the gradual construction of osteochondral organoids, starting at the bottom and going all the way to the top. Cell Sources: The basis of this strategy involves the selection of appropriate cell types, such as bone marrow mesenchymal stem cells, human umbilical cord mesenchymal stem cells, induced pluripotent stem cell-derived mesenchymal stem cells, induced pluripotent stem cells, osteoblasts, chondrocytes, and endothelial cells. Matrix Gel Selection: The next layer involves the selection of appropriate matrix materials to support cell growth and differentiation layer by layer. Commonly used matrices include matrix gels, collagen hydrogels, polyethylene glycol hydrogels, DNA hydrogels, and Construction Techniques: Key techniques such as layered induction, 3D printing, and microfluidics are employed to guide the development of osteochondral organoid structures. Directed Differentiation: Specific cytokines and mechanical interventions are essential to drive cell differentiation and tissue development within the organoids. Formation of fully developed osteochondral organoids at the apex. Image created by Figdraw.

An osteochondral organoid is a three-dimensional micro-osteochondral tissue constructed based on stem or progenitor cells. It possesses the properties of self-renewal and self-organization and is capable of mimicking the spatial structure of natural osteochondral units (Fatehullah et al., 2016). The construction of osteochondral organoids that possess physiological functions necessitates the coordinated regulation of numerous variables. These variables include cellular components, matrix gel materials, biofabrication techniques, and differentiation-inducing microenvironments (Figure 1). This biomimetic approach not only replicates tissue complexity but also drives innovation in regenerative medicine technologies.

### 2.1 Cell sources

**FIGURE 2:**
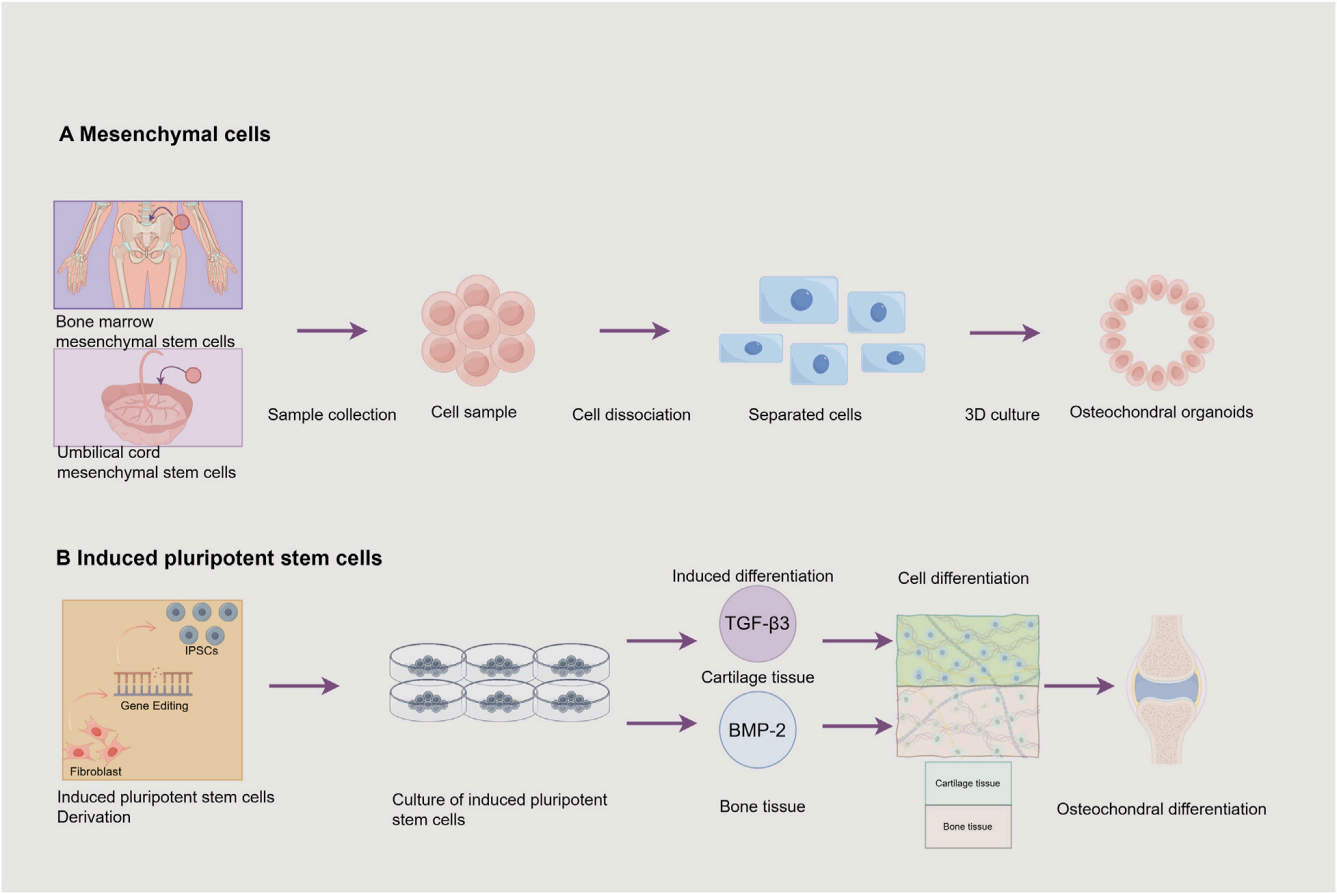
Cell Sources for Osteochondral Organoids. This figure illustrates 2 cell sources and their associated processes for constructing osteochondral organoids: **(A)** Mesenchymal cells: Mesenchymal stem cells can be obtained from the bone marrow and the umbilical cord. The process involves sample collection to obtain a cell sample, followed by cell dissociation to get separated cells. These cells are then cultured in 3D to produce osteochondral organoids. **(B)** Induced pluripotent stem cells: Fibroblasts are induced into induced pluripotent stem cells (iPSCs) through gene editing. Subsequently, the induced pluripotent stem cells are cultured. Through induced differentiation, transforming growth factor -β3 (TGF -β3) induces the formation of cartilage tissue, and bone morphogenetic protein −2 (BMP -2) induces the formation of bone tissue, ultimately achieving osteochondral differentiation. Image created by Figdraw.

The fundamental principle of osteochondral organoid technology relies on stem cells with multilineage differentiation potential. These stem cells are employed to construct 3D microstructures replicating native osteochondral tissue architecture. This requires precise spatiotemporal control of growth and differentiation to achieve biomimetic tissue formation (Wang J. et al., 2025). MSCs and iPSCs have become predominant cell sources for osteochondral organoids due to their exceptional plasticity and multilineage differentiation capacity (Urlić and Ivković, 2021) (Figure 2) (Table 1).

**TABLE 1:** Comparison of cell sources.

References	Cell source	Scaffold/Matrix	Culture method	Key features	Applications	Limitations
Lin et al. (2019)	IPSCs reprogrammed from hBM-MSCs	Methacrylated gelatin (mGL)-crosslinked hydrogel scaffold	Dual-flow bioreactor system with cartilage induction medium (top) and osteogenic induction medium (bottom)	Simulates cartilage-bone microenvironment; promotes functional cell-cell interaction; enables drug screening	Osteoarthritis modeling; drug development	Lacks tide line-like structure; simple scaffold composition; requires further validation of drug screening efficacy
Notoh et al. (2024)	MSCs	Basement membrane extract (BME, containing laminin, collagen IV)	3D culture: MSCs mixed with BME to form cartilage organoids; differentiation regulated via SMAD/NF-kB signaling pathways	Enhances cartilage maturation; upregulates chondrogenic genes; mimics endochondral ossification	Skeletal development research; bone defect repair	High batch variability in BME; challenges in modeling vascular invasion; limited control over terminal ossification
Yang et al. (2023b)	hUC-MSCs	CH-Microcryogels: hyaluronic acid [HA]-based; OS-Microcryogels: hydroxyproline [HYP]-based	Self-assembly into biphasic structures after 7-day pre-differentiation; mixed induction medium	Directed differentiation capacity; no layer separation; spontaneous *in vivo* assembly	Osteochondral regeneration; drug screening	Complex fabrication process; small sample size; translational challenges; potential immune reactions
Rodríguez Ruiz et al. (2022)	Primary chondrocytes from osteoarthritis patients; hiPSCs, with CCAL1 mutation and repair	3D culture matrix	TGF-β3-induced chondrogenesis of hiPSCs into cartilage organoids; BMP-2-driven osteogenic differentiation	Recapitulates pathological features (fibrosis, mineralization); demonstrates differential gene expression	Disease modeling; drug screening; mechanistic studies	Limited simulation of cell-cell crosstalk; donor-to-donor variability; low statistical power
Limraksasin et al. (2020)	Mouse iPSCs (reprogrammed from gingival fibroblasts)	No scaffold; ultra-low attachment microwell plates for embryoid body (EB) formation	Microspace aggregation into spherical constructs; shaking culture with stage-specific induction osteogenic/cartilage	Self-organization capacity; tunable bone/cartilage ratio; expression of lineage-specific markers	Regenerative medicine; disease modeling	Species-specific limitations; low structural complexity; unknown long-term stability
O’Connor et al. (2021)	Mouse iPSCs (reprogrammed from tail fibroblasts)	No scaffold; time-dependent growth factor exposure (TGF-β3 followed by BMP2)	Micromass culture with sequential growth factor induction to mimic endochondral ossification	Models endochondral ossification; multi-gene expression validation; multipotent differentiation capacity	Osteoarthritis modeling; personalized medicine	Limited redifferentiation potential; complex growth factor titration; insufficient *in vivo* validation

Notes: hUC-MSCs, Human umbilical cord-derived mesenchymal stem cells; hiPSCs, human induced pluripotent stem cells.

#### 2.1.1 Mesenchymal stem cells

Mesenchymal stem cells (MSCs) offer distinct advantages for osteochondral organoid construction through their multilineage differentiation capacity and paracrine signaling (Zhu et al., 2024). BMSCs modulate inflammatory microenvironments via exosome and miRNA secretion, attenuating cartilage degeneration and promoting tissue regeneration (Wang et al., 2022). BMSC-derived organoids are significantly influenced by inflammatory microenvironments, with their therapeutic efficacy closely dependent on microenvironmental regulation (Zhang et al., 2025). Notoh et al. (2024) developed a BMSC-based cartilage-like organoid using basement membrane extract (BME), though BMSC acquisition shows donor dependence. BMSC-derived organoids demonstrate an extracellular matrix composition closer to native cartilage, rich in type II collagen and proteoglycans (Noh et al., 2023). However, this matrix exhibits progressive degradation over time. Notably, BMSCs display a strong tendency toward hypertrophic differentiation during chondrogenesis, often leading to calcification and ossification (Alahdal et al., 2021). Studies consistently detect hypertrophy markers in BMSC-derived chondrogenic organoids, potentially compromising their long-term therapeutic efficacy (Zhang et al., 2021). While BMSCs possess strong expansion capacity, they exhibit significant batch-to-batch variability—particularly influenced by donor age and osteoarthritis disease progression (Zupan and Stražar, 2024).

Compared to BMSCs, human umbilical cord-derived MSCs (hUC-MSCs) demonstrate superior chondrogenic potential in 3D culture, forming cartilage organoids with enhanced regenerative capacity (Zhang Y. et al., 2023). hUC-MSCs exhibit greater clonogenicity, proliferation rate, migratory potential, and immunomodulatory activity, along with increased secretion of pro-chondrogenic factors (An et al., 2025). Additionally, preclinical studies suggest that UC-MSCs have a lower risk of hypertrophy compared to BM-MSCs (Chen X. et al., 2024). Their standardized sourcing and robust expansion capacity further support scalability for clinical applications. Researchers achieved osteochondral organoid self-assembly via spatially controlled differentiation of umbilical cord MSCs in engineered microgels, advancing hierarchical tissue regeneration, though human umbilical cord MSCs require neonatal umbilical cord as a source, which is limited by time and geographic location (Yang Z. et al., 2023).

Conventional tissue-derived MSCs often face challenges such as osteogenic differentiation and limited expansion capacity (Hamilton et al., 2023). In contrast, iPSC-derived MSCs (iPSC-MSCs), generated through standardized differentiation protocols, offer a more stable and reproducible cell source. Compared to primary MSCs, iPSC-MSCs exhibit superior proliferative capacity, enhanced immunomodulatory function, and higher biological efficiency (Shi et al., 2024). In preclinical studies, iPSC-MSCs and their derivatives successfully integrated into damaged joints in both rabbit ACLT (anterior cruciate ligament transection) models and primate cartilage defect models, promoting long-term tissue repair without immune rejection (Abe et al., 2023; Kim J. et al., 2024). The differentiation protocol was established to generate osteochondral organoids from hiPSC-derived MSCs, with the demonstration that osteoprotegerin (OPG) mutations disrupt mineralization processes in both cartilage and bone compartments of the organoids (Rodríguez Ruiz et al., 2022). This model provides new insights into the understanding of the pathological mechanisms of osteoarthritis and other related diseases. Given their scalability, functional superiority, and compatibility with organoid systems, iPSC-MSCs represent a promising tool for OA therapy and cartilage tissue engineering.

These differences suggest that hUC-MSCs may be more suitable for constructing therapeutic cartilage organoids, while BM-MSCs are better suited for disease mechanism research. Currently, iPSC-MSCs primarily serve as a tool for basic research, as their clinical application requires further safety validation. When selecting a tissue source, key considerations include cell accessibility, expansion capacity, and specific therapeutic objectives.

#### 2.1.2 Induced pluripotent stem cells

Induced pluripotent stem cells (iPSCs) are generated through genetic reprogramming of somatic cells to acquire embryonic stem cell-like properties, including self-renewal capacity and multilineage differentiation potential. iPSCs have been widely applied in regenerative medicine, disease modeling, and drug discovery, particularly showing promise for osteochondral organoid construction and articular cartilage repair (Abe et al., 2023; Liuyang et al., 2023; An et al., 2024). In 2019, Lin et al. developed a microphysiological osteochondral chip based on iPSCs and mimicked the pathologic alterations of OA, laying a foundation for the subsequent development of osteochondral organoid (Lin et al., 2019). In 2020, Limraksasin et al. generated mouse iPSC-derived osteochondral organoids using microwell and dynamic culture systems, offering novel construction strategies (Limraksasin et al., 2020). Mouse iPSC-derived osteochondral organoids were developed through timed exposure to TGF-β3 and BMP2 in 2021, effectively modeling endochondral ossification (O’Connor et al., 2021). While iPSC technology provides unlimited cell sources for organoid construction, current high costs limit widespread application and require future cost-reduction strategies.

### 2.2 Matrix gels

Hydrogel matrices serve as essential 3D scaffolds for osteochondral organoid engineering. Hydrogels are classified as natural or synthetic based on their origin and regulatory mechanisms, each offering distinct advantages in bioactivity, mechanical strength, and responsiveness. Combining the bioactivity of natural hydrogels with the programmability of synthetic ones enables precise modulation of the cellular microenvironment. Biomimetic designs have advanced from single-material systems to multifunctional platforms with enhanced performance.

#### 2.2.1 ​​Natural hydrogels​​

Presently, Matrigel remains the gold standard hydrogel for osteochondral organoid culture owing to its unique bioactive properties. This matrix can effectively support the adhesion, viability and expansion of stem cells. Its rich collagen composition activates key signaling pathways that direct MSC differentiation toward osteogenic and chondrogenic lineages. This facilitates *in vitro* generation of osteochondral organoids with native-like histoarchitecture and functionality. However, Matrigel’s undefined composition, batch variability, and murine tumor origin limit its applications (Kaur et al., 2021). These defects not only hinder the experimental reproducibility of the technology, but also impose significant limitations on its clinical translation. Consequently, developing standardized hydrogels with defined compositions has become a research priority.

Furthermore, Collagen hydrogels represent clinically promising biomaterials that combine native ECM bioactivity with tunable physical properties. Its three-dimensional network structure can accurately mimic the multilayered fibrous architecture of osteochondral tissues, and the unique arginine-glycine-aspartic acid (RGD) sequence endows it with excellent cell adhesion properties (Liu et al., 2019). Recent advances demonstrate collagen hydrogels’ potential as Matrigel alternatives for osteochondral regeneration. It has also exhibited a remarkable capacity to meticulously modulate stem cell differentiation in osteochondral organoid cultures.

#### 2.2.2 ​​Synthetic hydrogels​​

While natural hydrogels effectively mimic native tissue ECM and offer good biocompatibility, their clinical translation is constrained by batch variability and undefined compositions. Synthetic hydrogels have emerged as promising alternatives due to their reproducible properties and tunable biofunctionality (Chen W. et al., 2024) (Table 2). PEG’s bioinert nature and customizable modification sites make it ideal for osteochondral applications. Li et al. developed piezoelectric GelMA/PEG-BT nanocomposite hydrogels that transduce low-intensity pulsed ultrasound (LIPUS) into endogenous electrical cues, activating Wnt/β-catenin and PI3K/Akt pathways to enhance osteogenesis and bone defect healing (Li M. et al., 2025).

**TABLE 2:** Summary of composite hydrogel scaffolds.

Reference	Scaffold Structure	Loaded Cells	Key Innovations	Potential Applications	advantages
Ni et al. (2020)	SF/HPMC-MA dual-network hydrogel (β-sheet SF + UV-crosslinked HPMC-MA)	BMSCs	1. Dual-network structure enhances mechanical properties 2. Low-power ultrasonic induction of β-sheet formation 3. Promotes BMSCs proliferation and chondrogenic gene expression	Cartilage tissue repair	1. Excellent mechanical properties and biocompatibility 2. Precise 3D printing capability 3. Facilitates chondrogenic differentiation
Yang et al. (2023a)	PEMN hydrogel (agar-based, pre-shaped + post-osmotic enhancement)	BMSCs	1. Physical crosslinking for high toughness 2. No chemical crosslinkers required 3. Controllable shape 4. Induces endogenous cell mineralization	Osteochondral regeneration, drug delivery	1. High mechanical strength and degradability 2. Precise control of complex shapes 3. Strong adhesiveness for tissue integration 4. Promotes bone regeneration
Xiao et al. (2025)	HG-AA1:1-SDF-1 composite hydrogel (HA-CHO/GeIMA + Arg-CDs + SDF-1α + Ca^2+^)	BMSCs, HUVECs	1. Acid-responsive release of SDF-1α to recruit endogenous BMSCs 2. Arg-CDs metabolize to generate NO, activating osteo/angiogenic pathways 3. Eliminates need for exogenous cell transplantation	Bone defect repair, orthopedic implants	1. Intelligent dual-controlled drug release 2. Synergy between bone formation and angiogenesis 3. Avoids risks of exogenous cells 4. Improves acidic microenvironment
Li et al. (2025a)	Gel/PBT@BMSCs piezoelectric hydrogel (GeIMA + PEG-modified BT nanoparticles)	BMSCs	1. 3D-printed barium titanate scaffold2. Combined with LIPUS to enhance piezoelectric effect and activate PI3K/Akt pathway 3. High-precision DLP printing	Bone tissue engineering, personalized medicine	1. Efficient bone regeneration 2. Balance of biocompatibility and mechanical strength 3. Customizable complex structures
Liu et al. (2024a)	Bilayer piezoelectric-conductive hydrogel (upper dECM-FF peptide piezoelectric layer + lower PEDOT/Gel-C conductive layer)	BMSCs	1. Combination of piezoelectricity and conductivity to mimic physiological properties 2. FF peptide self-assembly enhances performance 3. Mechanical-electrical stimulation coupling for directional differentiation	Osteoarthritis, sports injury repair	1. Biomimetic bilayer structure 2. Stable electrical output performance 3. Promotes cell migration and differentiation 4. High potential for clinical translation
Ma et al. (2023)	Magnetic composite microcarriers (dopamine-Fe_3_O_4_ porous structure)	BMSCs	1. Magnetic-responsive targeted localization 2. Static magnetic field promotes proliferation/differentiation 3. Porous structure supports cell growth	Cartilage regeneration, magnetic-targeted therapy	1. Magnetically controlled precise delivery 2. Enhances collagen secretion and cartilage maturation 3. Reduces postoperative pain 4. Excellent blood compatibility
Rong et al. (2024)	Composite hydrogel scaffold (GMHA + hollow magnetic hydroxyapatite microspheres)	BMSCs	1. Porous magnetic microspheres loaded with VEGF 2. Fe_3_O_4_ nanoparticles enhance osteogenic differentiation 3. Photopolymerized GMHA matrix	Subchondral bone repair	1. Efficient VEGF delivery 2. Accelerates new bone formation 3. Fine-tuned design for bone repair requirements

Notes: HUVECs, Human Umbilical Vein Endothelial Cells.

DNA hydrogels utilize DNA molecules as primary building blocks, forming a highly porous three-dimensional network through physical entanglement or chemical crosslinking (Mo et al., 2021). DNA hydrogels represent a novel class of 3D programmable biomaterials with sequence-specific self-assembly capabilities, offering unique advantages in biocompatibility, molecular recognition, and stimuli-responsiveness (Qiao et al., 2024). Zhu et al. engineered GelMA/DNA hybrid hydrogels that recapitulate both the biochemical and viscoelastic properties of native bone ECM, facilitating the self-organization of mineralized bone-like tissues (Zhu et al., 2025). RGD-functionalized silk fibroin/DNA (RSD) microspheres were designed to promote chondrogenesis via integrin-mediated mechanotransduction and enhanced GAG synthesis (Shen et al., 2024). These advances establish DNA-based hydrogels as a platform technology for osteochondral organoid engineering, combining molecular programmability with tissue-specific bioactivity to advance regenerative strategies.

Different types of hydrogels play crucial roles in promoting the self-organization of bone-cartilage zonal structures due to their unique biochemical and mechanical properties. Gradient hydrogels accurately mimic the biochemical and mechanical gradients found in native tissues, from superficial cartilage to deep bone, enabling zone-specific cell behavior and differentiation while maintaining paracrine signaling between cells (Zhu et al., 2023). Bilayer and multilayer hydrogels support dual-lineage differentiation of cartilage and bone cells through physical layering and functional delivery of growth factors (Wei et al., 2023; Yao H. et al., 2023). Composite hydrogels, which incorporate microgels or stem cell spheroids, enhance zonal control and interface integration (Lee et al., 2024). Smart hydrogels offer novel solutions for minimally invasive repair of irregular defects and interface stabilization (Zhang L. et al., 2023). Acellular hydrogels demonstrate significant translational potential, attributable to their inherently low immunogenicity and remarkable host cell recruitment capacity (Schwab et al., 2023).

The mechanical properties of hydrogels, such as stiffness and elastic modulus, finely regulate chondrogenic and osteogenic differentiation (Liu et al., 2023). Lower stiffness promotes dynamic cytoskeletal remodeling in chondrocytes and cartilage formation, whereas higher stiffness favors bone formation (He et al., 2025; Tong et al., 2025). Dynamic mechanical characteristics, including viscoelasticity and stress relaxation, further influence cell fate through mechanotransduction (Kim H.-S. et al., 2024). The key to achieving efficient functional integration of bone and cartilage lies in the rational design of biochemical gradients and mechanical zoning, combined with interfacial chemical adhesion and dynamic regulation strategies.

While hydrogel technology demonstrates significant promise for osteochondral organoid engineering, key translational challenges remain: batch-to-batch inconsistency in natural hydrogels, long-term biocompatibility concerns with synthetic polymers, and limited vascular/neural network integration. Future development should focus on AI-driven material optimization, integrated 3D bioprinting-organ-on-chip platforms, and patient-specific disease modeling for clinical translation. Advanced bioinspired hydrogel systems, through their tunable properties and multifunctional design, offer transformative potential for precision tissue engineering and personalized regenerative therapies.

### 2.3 Advanced biofabrication technologies​

#### 2.3.1 3D bioprinting

Scaffold-free organoids, which rely on cellular self-assembly, better recapitulate native tissue microarchitecture and are particularly suited for disease modeling (Luo et al., 2023). However, they face challenges in scalability and vascularization, often requiring external perfusion systems for adequate nutrient supply (Gao et al., 2025). 3D bioprinting enables precise spatial patterning to engineer complex tissue architectures with integrated vascular networks, while supporting high-throughput and reproducible organoid production (Hu et al., 2025). This has driven growing adoption of 3D bioprinting for organoid engineering (Su et al., 2021). 3D bioprinting precisely recapitulates articular cartilage’s hierarchical structure through controlled bioink deposition and multiscale scaffold fabrication (Matai et al., 2020; Banerjee et al., 2022). In 2018, Zhao et al. first combined computational modeling with airflow-assisted 3D bioprinting to generate vascularized bone organoids (Zhao et al., 2018). In this study, BMSCs and HUVECs were encapsulated in hydrogel microspheres and co-differentiated toward osteogenic and vascular lineages. Vascularized bone tissue formed within 10 days, which provides a solution to the difficult problem of vascularization of osteochondral organoids. In 2020, Ni et al. developed 3D-bioprinted BMSC-laden filipin-HPMC dual-network scaffolds that enhanced cartilage repair (Ni et al., 2020). In 2022, Zhang et al. fabricated 3D-bioprinted hMSC-seeded graphene oxide scaffolds (Zhang et al., 2022a). Cyclic mechanical loading in bioreactors enhanced the scaffolds’ mineralization, stiffness, and osteogenic potential. These results confirm mechanical stimulation’s importance in bone regeneration and advance functional osteochondral organoid development. In 2024, A GelMA/AlgMA/HAP hybrid bioink was developed by Su et al. to mimic the native bone ECM via innovative biomaterial integration (Wang J. et al., 2024). Using DLP bioprinting, they created biomimetic bone constructs supporting long-term culture and multilineage differentiation. The resulting microtissues exhibited native-like structure-function relationships and enhanced defect repair, advancing osteochondral regeneration.

Despite its advantages, 3D bioprinting faces challenges in controlled factor release, ECM homogeneity, and mechanical stability. Post-implantation structure-function relationships require systematic evaluation. Patient-specific cell variability poses additional challenges for consistent organoid functionality and clinical translation. Future integration with dynamic culture systems could enhance tissue maturation and microenvironmental adaptation. Therefore, the optimal strategy depends on the research objectives: scaffold-free approaches are preferable for investigating self-organization mechanisms or streamlined culture processes, hydrogel-based systems better suit studies requiring microenvironment control or dynamic culture conditions, while bioprinting offers advantages for applications demanding high spatial precision or vascular network integration.

The emergence of 4D bioprinting introduces temporal control to 3D-printed constructs, enabling dynamic shape-morphing and functional adaptation (Lai et al., 2024; Yarali et al., 2024). Smart materials with time-dependent properties can be programmed to respond to physiological cues, facilitating host integration of implanted osteochondral organoids (Annan et al., 2024). This approach achieves spatiotemporal coordination between structural remodeling and functional maturation, advancing regenerative medicine toward dynamic, patient-specific therapies.

#### 2.3.2 Microfluidic chip technology​​

Static culture, performed using standard Petri dishes or scaffold systems, works well for basic research and cost-effective setups. However, it often faces limitations in oxygen and nutrient distribution, which can lead to hypoxic conditions developing in the central areas of cell masses or tissues (Patel et al., 2021). Dynamic culture systems enhance nutrient/oxygen delivery while promoting cell proliferation, ECM deposition, and tissue functionality—better replicating physiological microenvironments *in vivo* (Li Y. et al., 2025). Microfluidic systems offer superior platforms for cellular studies and pharmaceutical development through precise microscale engineering (Bhatia and Ingber, 2014; Tolabi et al., 2023; Liu et al., 2024b). These platforms incorporate engineered microchannels and semipermeable membranes that: Support multicellular coculture systems, Recapitulate physiological tissue interfaces and mechanoenvironments. Consequently, they achieve more physiologically relevant tissue and organ functionality. Research has demonstrated that Microfluidic Hydrogel-Based Scaffolds (MHBS) can meticulously modulate the local concentration distribution of nutrients, oxygen gradients, and biochemical factors to facilitate the differentiation of stem cells into cartilage cells (Tolabi et al., 2023). Microfluidic-based osteochondral organoid systems offer three key advantages: (1) real-time microenvironment modulation, (2) enhanced culture reproducibility, and (3) scalable automated production. The microfluidic platform, which is based on Organ-on-a-Chip (OOC), dynamically simulates the biophysical and chemical signals of cartilage (Skoracka et al., 2024). This enables controlled investigation of OA pathogenesis and optimization of chondrogenic differentiation protocols. In addition, Quintard et al. engineered an innovative microfluidic-based microphysiological system featuring a functional endothelial network that integrated with mesenchymal spheroids, pancreatic islets, and iPSC-derived vasculature to establish perfusable vascular connections (Quintard et al., 2024). The vascularization approach markedly enhanced organoid growth kinetics, structural maturation, and physiological functionality. These results indicate its promising potential for resolving vascularization challenges in osteochondral organoid engineering.

The microfluidic system enhances the physiological relevance of osteochondral organoids by simulating hypoxic cartilage regions and normoxic bone regions (González-Guede et al., 2024). Osteochondral tissue chips can apply tissue-specific compression levels to sustain tissue viability and compositional stability for up to 2 months, and replicate the mechanical strain gradients of the joint microenvironment (Mainardi et al., 2025). Moreover, integrating biopolymers and decellularized extracellular matrix (dECM) as bio-ink can improve the density and cell distribution of cartilage ECM, thereby enhancing the accuracy of pathological simulation (Upadhyay et al., 2024). Microfluidic technology can achieve high-throughput screening through automated design, such as rapid sorting of MSC subsets with chondrogenic potential, significantly enhancing repair efficiency (Yang et al., 2024). Another study developed a joint chip containing cartilage and synovial compartments, supporting parallel testing of personalized therapies (Petta et al., 2024).

Microfluidic multi-tissue integration technology represents a pioneering approach in osteoarthritis research. The co-culture system leverages microfluidic platforms to simultaneously cultivate human osteoblasts, chondrocytes, fibroblasts, and macrophages, maintaining cell viability for up to 24 h and replicating joint environments in both healthy and diseased states (Mirazi and Wood, 2025). Additionally, a microphysiological system incorporating biphasic bioreactors enables signal transduction between cartilage and bone-like analogs, eliciting inflammatory responses in cartilage region (Smith et al., 2025). The joint-on-chip model, integrating cartilage and synovial compartments, employs hydrogel-embedded chondrocytes and synovial fibroblasts to evaluate personalized therapeutic strategies (Petta et al., 2024). Microfluidic technology further replicates the three-dimensional architecture of the synovium and associated vasculature, providing a physiologically relevant research model (Thompson et al., 2023). Moreover, the multi-region suspension tissue model, utilizing open microfluidic patterning (STOMP), generates suspended multi-region tissues that mimic natural interfaces, offering innovative tools for studying cell contraction and tissue integration (Haack et al., 2025). Collectively, these technologies underscore the transformative potential of microfluidic multi-tissue integration in advancing osteoarthritis research.

Despite its transformative potential in osteoarthritis research, microfluidic technology faces several challenges. The technical complexity of integrating multilayer scaffolds, such as bone-cartilage interfaces, hinders stable material adhesion and mechanical compatibility (Maherani et al., 2024). Additionally, current models struggle to replicate the high peripheral strain and dynamic mechanical environment of joints, while the dense cartilage extracellular matrix in chips restricts cell proliferation and microscale remodeling (Liu et al., 2024c). Furthermore, the modular design of organ-on-chip systems lacks standardized protocols and systematic integration of variables, such as sex and age (Conceição et al., 2025). These limitations impede the widespread adoption and accurate simulation of microfluidic technology in osteoarthritis studies.

#### 2.3.3 Integrated 3D bioprinting-microfluidics systems**​**

In a previous study, Davoodi et al. engineered biomimetic tissues replicating native tissue architecture and function through integrated extrusion bioprinting-microfluidics approaches (Davoodi et al., 2020). Microfluidic platforms enable pre-bioprinting optimization of culture parameters through controlled mechanical stimulation and gradient factor exposure. Subsequent precise cell-biomaterial assembly yields biomimetic tissue constructs. High-precision bioprinting generates stratified cartilage-bone interfaces (Lopa et al., 2018). Modular hydrogel bioinks incorporating chondrocyte-laden microspheres were developed to print scaffolds that recapitulate the hierarchical structure of articular cartilage (Yin et al., 2023). This highlights the therapeutic potential of integrated microfluidics-bioprinting systems for cartilage repair. Multi-material bioprinting allows for the creation of precisely patterned cellular compartments and channels (Wang T. et al., 2025). When combined with microfluidic control systems, it enables directional flow regulation (perpendicular or parallel to scaffolds) to guide cell distribution, on-chip spatiotemporal control of biochemical gradients, and precise 3D spatial organization of multiple cell types (Xu et al., 2025). The convergence of bioprinting, microfluidics and organoid technologies can better emulate chondrocyte niches, advancing functional cartilage regeneration.

### 2.4 Directed differentiation​​

Osteochondral organoid construction centers on chondrogenic and osteogenic differentiation - critical processes for OA reversal and cartilage defect repair (Dönges et al., 2024; Kronemberger et al., 2025). TGF-β/BMP signaling and mechanical cues coordinately regulate osteochondral organogenesis, enhancing stem cell differentiation toward chondrocyte and osteocyte lineages. TGF-β3 induced self-assembled spheroids develop articular cartilage-like morphology and molecular signatures. These spheroids simultaneously upregulate cartilage markers (COL2A1, ACAN). This established a framework for reproducible osteochondral organoid generation (Negishi et al., 2024). TGF-β/BMP signaling enhances both chondrogenic differentiation efficiency and cartilage matrix biomechanics. This occurs through upregulated proteoglycan and COL2 synthesis, improving organoid repair capacity (Kronemberger et al., 2025). Mechanical microenvironments regulate chondrocyte phenotype and ECM homeostasis via integrin-mediated mechanotransduction and ECM remodeling feedback loops. These mechanisms enhance the therapeutic potential of osteochondral organoids for cartilage repair (Ni et al., 2020).

## 3 Osteochondral organoids for osteoarthritis modeling

### 3.1 ​Pathomimetic modeling of OA using osteochondral organoids

**FIGURE 3:**
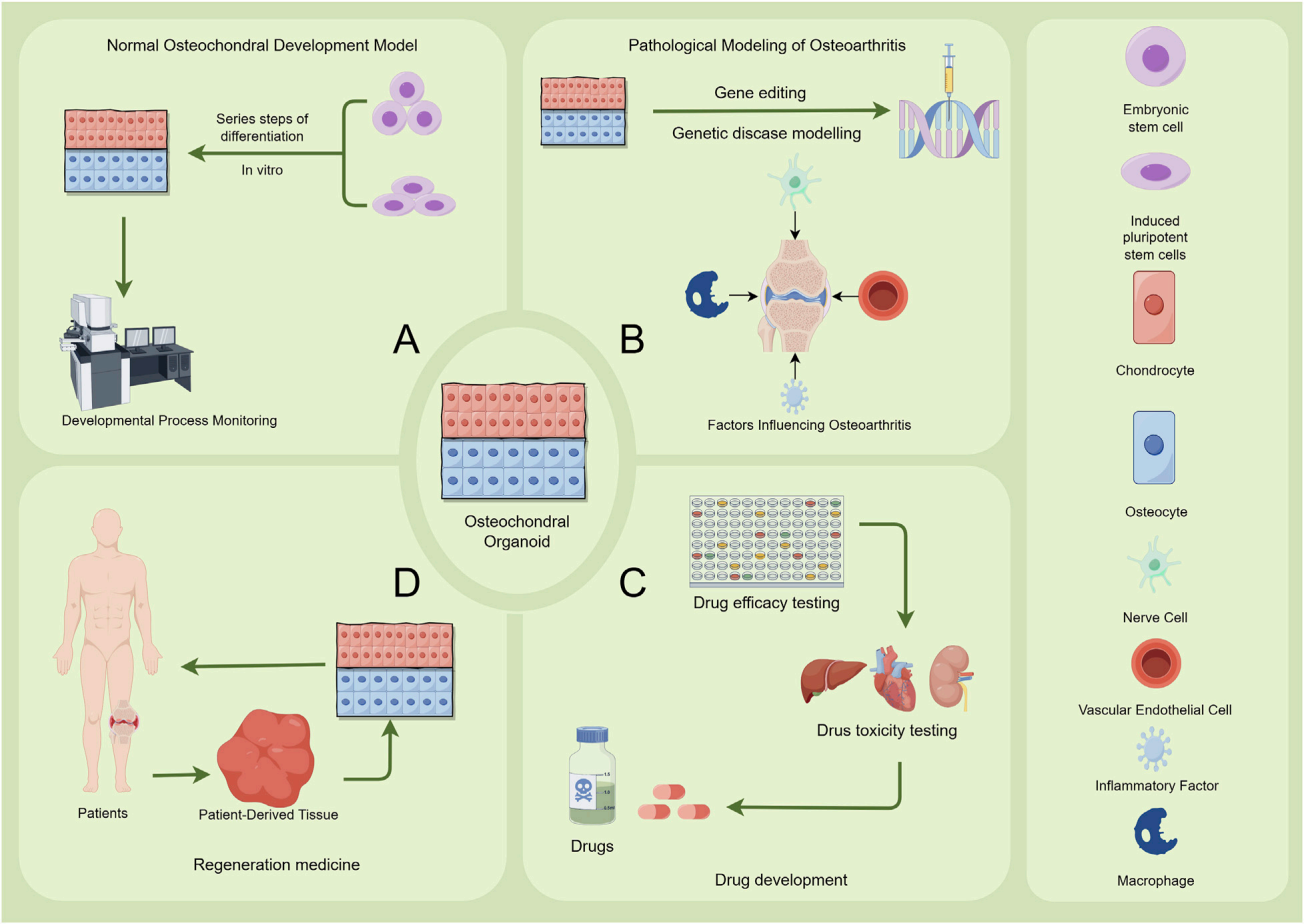
Applications of Osteochondral Organoids. The figure illustrates the applications of osteochondral organoids in four main areas: **(A)** Normal Osteochondral Development Model. Embryonic stem cells or induced pluripotent stem cells are utilized. Through a series of differentiation steps *in vitro*, an osteochondral structure is formed. Developmental Process Monitoring, such as microscopic observation, is employed to study the normal developmental process of osteochondral tissues. **(B)** Pathological Modeling of Osteoarthritis. Gene editing is used for genetic disease modelling. Additionally, relevant factors including nerve cells, inflammatory factors, vascular endothelial cells, and macrophages are introduced to investigate the factors influencing the onset and progression of osteoarthritis. **(C)** Drug Development Osteochondral organoids are used for drug efficacy testing and drug toxicity testing. These tests evaluate the therapeutic effects and potential toxicity of drugs for osteochondral - related conditions, providing data support for drug development. **(D)** Regeneration Medicine Patient-derived tissues are processed and cultured to generate organoids. Subsequently, patient-specific osteochondral organoids are utilized in regenerative medicine research and therapies to promote the repair and regeneration of damaged osteochondral tissues in patients. Image created by Figdraw.

Osteochondral organoids effectively mimic *in vivo* microenvironments, enabling detailed studies of cartilage degeneration and subchondral bone remodeling in OA (Figure 3). These models serve as dual-purpose platforms for both elucidating OA pathogenesis and developing diagnostic/therapeutic innovations. The iPSC-derived osteochondral organoids successfully repaired full-thickness cartilage defects in rabbit models, restoring biomechanical function via ECM-mediated host integration (Ali et al., 2024). Nevertheless, long-term functional outcomes require validation under physiologically relevant loading conditions. Van Hoolwerff et al. generated cartilage organoids from hiPSC-derived chondroprogenitors, revealing FN1-C518F mutations disrupt FN1-COL2 binding and induce OA-like chondrocyte phenotypes (van Hoolwerff et al., 2021). This finding identifies FN1-COL2 interactions as novel therapeutic targets for OA intervention. COL6A3 variants in hiPSCs were engineered via CRISPR-Cas9 by Bloks et al. for the purpose of modeling cartilage pathology (Bloks et al., 2024). COL6A3 variants disrupted mechanotransduction, causing cartilage matrix metabolic imbalance under mechanical stress. This process upregulates key inflammatory regulators (PTGS2, PECAM1, ADAMTS5, and lncRNA MIR31HG), providing insights into OA’s mechano-inflammatory pathways. Gene-edited organoids thus offer valuable tools for decoding OA’s molecular networks. However, Current models need improved dynamic loading systems and multi-tissue integration capabilities. Future development requires smart materials and multi-interface engineering to build pathomimetic models for clinical translation.

### 3.2 High-throughput drug screening platforms​​

Traditional bone tissue engineering relies on iterative *in vivo* testing to optimize scaffold composition, cell sources, and growth factors, but this approach is limited by high costs, lengthy timelines, and poor reproducibility due to non-standardized conditions (Horvath et al., 2016). Osteochondral organoids, as self-assembled 3D microtissues, faithfully recapitulate disease pathophysiology. Beyond personalized drug screening and regenerative therapy assessment, they serve as powerful tools for medical research (Zeng et al., 2023). Their biomimetic microenvironments enable OA molecular network studies and accelerate bench-to-bedside translation (Piraino et al., 2024). Abraham et al. developed OA-mimicking articular spheroids using osteochondral organoids to evaluate A2A adenosine receptor agonists, demonstrating their potential as high-fidelity platforms for pharmacological validation (Abraham et al., 2022). Integrated osteochondral organoid systems enable comprehensive evaluation of skeletotropic compounds and regenerative therapies while maintaining critical tissue crosstalk, bridging target validation to precision medicine. In preclinical studies, intra-articular Sema3A administration in murine and non-human primate OA models inhibited aberrant innervation and suppressed hypertrophic chondrocyte markers, thereby maintaining joint homeostasis (Huang et al., 2025). Clinical trials confirmed Sema3A’s ability to alleviate pain and slow OA progression, supporting organoid-based screening of Sema3A-related therapies. Future integration of high-throughput drug screening and Sema3A-upregulating gene editing with OA organoids may yield breakthrough therapies.

However, current osteochondral organoid models primarily focus on isolated bone or cartilage tissues, limiting their ability to simulate multi-tissue interactions involving the synovium, blood vessels, and nerves. This constraint reduces the accuracy of drug response and metabolite toxicity predictions. Future advancements should prioritize multi-tissue organoids integrated with microarray platforms to model dynamic inflammatory networks. Additionally, incorporating hepatic metabolism and renal clearance functions is essential for accurate drug metabolite toxicity evaluation. Co-culturing osteochondral organoids with liver organoids could establish comprehensive drug metabolism platforms. To address challenges in patient-derived organoids, such as prolonged timelines, high costs, and donor variability, integrating single-cell sequencing with standardized patient subtype-response databases offer a promising solution. This approach supports data-driven, personalized treatment strategies.

### 3.3 Regenerative medicine and tissue repair strategies​​

#### 3.3.1 ​​Osteochondral organoid transplantation​

Current clinical management of advanced OA and large cartilage defects relies on autologous or allogeneic cartilage transplantation. However, autografts face donor site morbidity and supply limitations, while allografts carry risks of immune rejection and pathogen transmission. Osteochondral organoids present a promising alternative addressing these challenges. Tam et al. first demonstrated stable cartilage formation through heterotopic transplantation of engineered osteochondral organoids (Tam et al., 2021). This work establishes that IL-1β disrupts bone repair via MMP13-mediated ECM degradation, identifying a key molecular target for restoring the inflammation-regeneration equilibrium. While organoid models demonstrate promising cartilage-to-bone conversion rates and repair efficiency *in vitro*, their structural integrity under physiological loading conditions and functional integration with native tissues require rigorous preclinical validation. Researchers developed single chondral organoid approach derived from bone marrow mesenchymal stem cells (BMSCs) mimicking native tissue architecture and biomechanics (Chen et al., 2025). Under the guidance of the natural microenvironment at the osteochondral defect site, heterogeneous osteochondral regeneration with a precise gradient can be achieved, which represents an important advancement for clinical applications. Osteochondral organoids recapitulate endogenous cartilage repair mechanisms. Moreover, scalable production permits direct implantation of large-format organoids for enhanced regeneration. This breakthrough establishes new paradigms for orthopedic regenerative medicine.

#### 3.3.2 Cellular microenvironment

After implantation, osteochondral organoids act as therapeutic agents, with the injury microenvironment playing a pivotal role in successful tissue repair. Key factors influencing repair include hypoxia, inflammation, immune regulation, and signaling pathway interactions (Gu et al., 2023).

##### 3.3.2.1 ​Hypoxia-mediated regulation​​

Given the stark differential responses of cartilage and bone to oxygen tension, biomimetic oxygen gradients play a pivotal role in reconstructing the ​​osteochondral unit (OCU) (Taheem et al., 2020)​​. Dehghani et al. demonstrated that ​​hypoxic preconditioning​​ significantly enhances the chondrogenic differentiation of ​​buccal fat pad stem cells (BFPSCs)​​ in a bilayer chitosan hydrogel scaffold, improving osteochondral defect repair (Dehghani Nazhvani et al., 2021). This finding underscores the importance of oxygen gradient design in osteochondral regeneration and offers new insights for optimizing ​​organoid-based therapeutic strategies​​. Further supporting this, studies show that ​​hypoxia-preconditioned apoptotic extracellular vesicles (H-ApoEVs)​​ derived from mesenchymal stem cells (MSCs) are more effective than normoxic ApoEVs in promoting ​​stem cell migration, proliferation, and macrophage M2 polarization​​, thereby enhancing cartilage repair (Ding et al., 2024). By integrating ​​3D-printed ECM scaffolds​​ for mechanical support with ​​biomimetic oxygen microenvironments​​, this delivery system presents a novel approach for ​​exosome-mediated cartilage regeneration​​.

Additionally, research indicates that ​​bone marrow-derived MSCs (BMSCs)​​ expanded under normoxia progressively lose stemness, whereas ​​hypoxic preconditioning​​ preserves their undifferentiated state by ​​suppressing oxidative stress and activating the HIF-1α signaling axis​​, thereby improving regenerative efficacy (Camarero-Espinosa et al., 2024). Intriguingly, selective activation of the ​​TGF-β pathway​​ can simultaneously support ​​stem cell quiescence maintenance​​ and ​​lineage-specific differentiation​​ in a single culture system, suggesting that hypoxia may dynamically balance cell fate decisions by modulating ​​HIF-1α–SMAD crosstalk​​ (Yao L. et al., 2023). These findings collectively suggest that ​​hypoxic preconditioning​​ should be incorporated into future ​​osteochondral organoid models​​ to enhance cartilage repair outcomes.

##### 3.3.2.2 ​Inflammation and immunomodulation​​

Inflammation and immunomodulation exert dual-phase regulation during cartilage repair, balancing pro-regenerative and anti-inflammatory responses. Activin A, a member of the transforming growth factor-beta (TGF-β) superfamily, exhibits significant upregulation during the process of fracture healing. It directly promotes the differentiation of fibroblasts, chondrocytes, and osteoblasts through the activation of the ACVR2B receptor, while remaining virtually silent in intact bone (Yao L. et al., 2023). The TGF-β family regulates diverse processes including chondrogenesis via SOX9 activation (Camarero-Espinosa et al., 2024). It also mediates immune tolerance by polarizing M2 macrophages and enhancing Treg function, maintaining repair-phase immune homeostasis. Optogenetic tools enabled spatiotemporally precise TGF-β pathway control, effectively correcting cartilage defects and bone remodeling disorders (Wu et al., 2023). These findings highlight the potential of integrating inflammatory-immune balance mechanisms into next-generation osteochondral organoids. For example, precise control of Activin A/TGF-β signaling pathway expression can be achieved by mimicking the osteochondral repair microenvironment. Optogenetic TGF-β release could promote M2 macrophage polarization and Treg activation, enhancing immune homeostasis at the cartilage-bone interface for advanced *in vitro* modeling. Dynamic hydrogels with timed-release properties could enable biomimetic platforms that support both chondrogenesis and immune tolerance. These models may address clinical challenges in balancing tissue regeneration and inflammation during osteochondral repair.

## 4 Current challenges and future perspectives

### 4.1 Technical bottlenecks ​​

The challenge of vascularization of osteochondral organoids during long-term culture is directly related to functional maintenance and graft survival (He et al., 2022). Experiments have shown that organoids lacking functional vascularization have significantly reduced viability after 4 weeks of *in vitro* culture, with up to 60% attenuation of their secretory function (Abraham et al., 2024). *In vitro* cartilage organoid culture can benefit from the introduction of vascular endothelial growth factor (VEGF), which promotes organoid hypertrophic differentiation. However, this requires regulation through anti-angiogenic drugs like Axitinib to prevent ectopic ossification (Vanlauwe et al., 2024). Cord blood endothelial colony forming cells (CBECFCs) have been shown to form dynamic vascular networks with inflammatory responsiveness due to their strong angiogenic capacity and immunomodulatory properties (Smadja et al., 2025). Coculture of CBECFCs with organoids facilitates stable capillary network formation in engineered bone regions while simultaneously enhancing chondrogenic and osteogenic differentiation. Microfluidic chip technology can replicate the early vascular networks of endochondral ossification, providing a model for studying human endochondral ossification (Kesharwani et al., 2025). Alternatively, bioprinting can be used to directly print endothelial cell channels to generate vasculature (Cadena et al., 2024).

Immune rejection presents a major translational hurdle for osteochondral organoids. As three-dimensional cell cultures, organoids retain the characteristics of their tissue of origin, which may trigger immune rejection during transplantation. Studies have shown that fully xenogeneic renal organoids exhibit stronger rejection responses than chimeric organoids (Elder et al., 2018). CRISPR-Cas9-mediated knockdown of HLA class I molecules effectively reduces T-cell-mediated immune recognition in osteochondral organoids but may compromise their immunosurveillance function (Gaykema et al., 2024). This paradox is particularly prominent in osteochondral Organoid - the unique immune immunity properties of chondrocytes contrast with the strong immunogenicity of osteoblasts. Recent studies have shown that combining the low immunogenicity characteristics of CBECFCs (cord blood endothelial colony-forming cells) with precise immunoediting techniques may allow the construction of general-purpose osteochondral Organoids with vascularization potential (Smadja et al., 2025). More notably, Joint-specific immune isolation devices incorporating TGF-β modulation could create multi-layered immune barriers for osteochondral organoids (Wang X. et al., 2025). This integrated approach minimizes systemic immunosuppression risks and enhances clinical translation. Additionally, during the construction of organoid-scaffold complexes, when engineered hydrogels are used as carrier matrices, fine surface functionalization modification or matrix component optimization can be employed to effectively regulate host immune responses and significantly reduce the risk of foreign body reactions triggered by material implantation (Chen et al., 2025). By mimicking the biochemical-mechanical microenvironment of the natural extracellular matrix, this strategy can simultaneously achieve the dual objectives of enhancing immunocompatibility and maintaining organoid function.

Organoids also present potential safety risks that require careful evaluation. Organoids pose infection risks post-implantation due to incomplete sterilization of their internal 3D structures. Moreover, cell migration to non-target areas may cause abnormal vascularization in cartilage regions, disrupting physiological function. iPSC-derived products risk containing residual undifferentiated iPSCs with high proliferative and differentiation potential, potentially forming teratomas or tumors (Dou et al., 2025). Studies show even trace iPSC residues pose risks, necessitating highly sensitive quality control methods or suicide gene switches (Hill et al., 2024).

Clinical translation of osteochondral organoids demands resolving key challenges in GMP standardization, regulatory clarity, and scalable production (Wang X. et al., 2025). GMP hurdles include complex workflows, batch variability, and inconsistent organoid stability from iPSCs/BMSCs due to inflammatory microenvironments (Zhang et al., 2025). Potential solutions involve AI-driven process optimization or bioprinting for precise heterostructure control (El-Tanani et al., 2025). Regulatory gaps persist, particularly for composite tissues (e.g., bone-cartilage integration) and limited clinical trial data. International consensus is needed to define organoid classification and approval pathways. Scalability barriers stem from high costs and fidelity limitations. Future efforts should prioritize non-viral vectors, modular production, large-animal validation, and standardized imaging evaluations (Hall et al., 2021; Sun et al., 2025).

### 4.2 ​​Emerging interdisciplinary directions​​

The integration of smart materials with organ-on-a-chip platforms has revolutionized OA and cartilage repair research. O'Donnell et al.'s GelMA-based 3D organ-chip maintains stem cell adipogenic potential, offering novel insights into knee OA pathogenesis (O’Donnell et al., 2020). Hydrogel-based microphysiological systems recapitulate OA microenvironments by co-culturing synovial fibroblasts and chondrocytes, enabling investigation of synovial macrophage accumulation and therapeutic discovery (Chijimatsu et al., 2017). Advanced tissue engineering enables microphysiological platforms that precisely control mechanical and biochemical gradients for drug screening and disease modeling.

Empowered by multi-omics technologies and spatial parsing methods, the depth and precision of organ-on-chip research will be significantly enhanced. The application of single-cell sequencing and spatial transcriptomics has enabled researchers to analyze the mechanisms of cellular heterogeneity and microenvironmental interactions within the microarray. Integrated flow cytometry-RNA sequencing revealed distinct pro-angiogenic profiles between BMMSCs and iPSCs-MSCs, informing optimized cell therapy approaches (Gonzalez-Rubio et al., 2025). The integration of such technologies not only enables dynamic tracking of disease-related gene expression changes, but also reveals the spatial and temporal characteristics of cell-matrix interactions at the micro-scale.

The introduction of external physical stimuli such as magnetic fields, optogenetics and mechanical forces may further expand the boundaries of osteochondral Organoid functionalization. Optogenetic preconditioning of monocytes suppresses inflammatory migration, demonstrating precise immune modulation capabilities (Chijimatsu et al., 2017). Microfluidic systems accurately replicate articular cartilage biomechanics, enabling mechanistic studies of mechanical signaling in OA pathogenesis and repair (Grottkau et al., 2022). Combined application of these technologies in organoid engineering will both elucidate OA pathophysiology and accelerate personalized therapy development.

**FIGURE 4:**
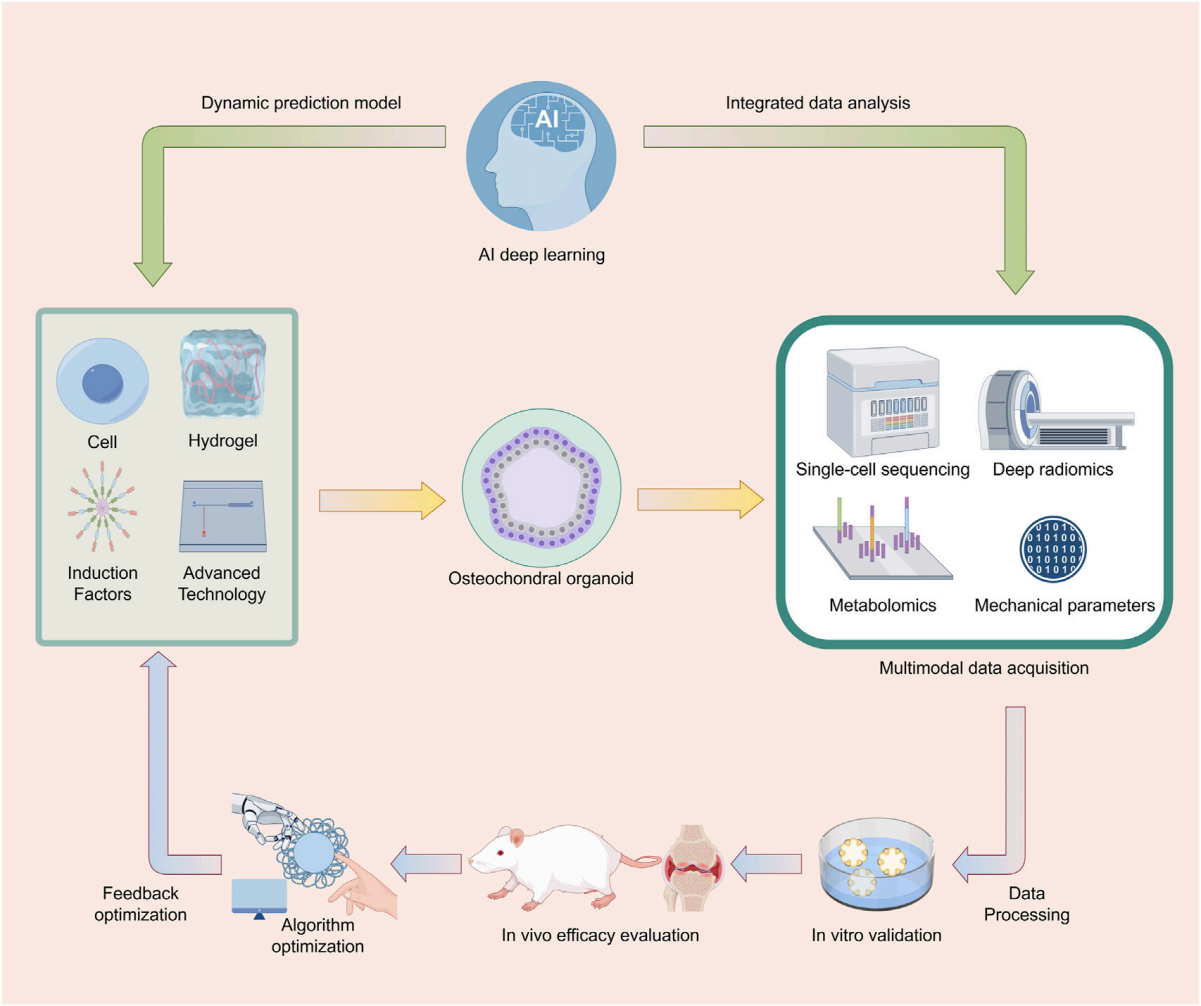
AI-Driven Cyclic Optimization System for Osteochondral Organoid Research. This figure illustrates a cyclic process for the research of osteochondral organoids using AI deep - learning technology. First, osteochondral organoids are constructed using cells, hydrogels, induction factors, and advanced technologies. Subsequently, multimodal data acquisition methods such as single - cell sequencing, deep radiomics, metabolomics, and mechanical parameters are employed to obtain data, which is then subjected to data processing. Following this, the organoids are studied through in - vitro validation and in - vivo efficacy evaluation. The data generated during the research process is integratively analyzed and then fed back to AI deep - learning to establish a dynamic prediction model. The model undergoes feedback optimization through algorithm optimization, further guiding the construction of osteochondral organoids, thus forming a cyclic and iterative research system to continuously optimize and advance research related to osteochondral organoids. Image created by Figdraw.

Artificial intelligence (AI) significantly advances osteochondral organoid research through applications in material optimization, organoid construction, data analysis, and disease modeling (Figure 4). In material optimization and scaffold design, AI employs computational Design of Experiments (DoE) methods to refine biomaterial parameters, such as those for silk fibroin (SF)-based hydrogels, enhancing biomimetic performance (Shen et al., 2025). This approach improves simulation of the vascularization gradient in osteochondral tissue while optimizing scaffold porosity and mechanical properties to closely mimic natural tissue structure and function (Corrado et al., 2025). In organoid construction and differentiation, AI-supported three-dimensional culture systems facilitate the differentiation of BMSCs into chondrocytes and osteoblasts, producing osteochondral organoids with gradient heterogeneity and enabling synchronous regeneration of cartilage and bone tissues post-implantation in animal models (Chen et al., 2025). Furthermore, AI-driven deep learning analyzes high-throughput organoid data, minimizing errors associated with manual analysis, accelerating disease mechanism elucidation and drug screening, simulating pathological processes in diseases like osteoarthritis, and enabling predictive models for personalized therapies (Maramraju et al., 2024; Yang et al., 2025). In the future, AI-based quantitative models for assessing inflammation, currently applied in cardiac organoid research, could be adapted for osteochondral organoid studies (Lin et al., 2025).

The convergence of organ chips, smart materials, physical modulation technologies, and multi-omics establishes an end-to-end framework bridging molecular mechanisms to clinical applications. This interdisciplinary approach delivers innovative tools for OA precision medicine while advancing foundational technologies for regenerative breakthroughs.

## 5 ​Conclusion

Osteochondral organoids represent a transformative paradigm in OA research, integrating advances in stem cell biology, biomaterials science, and biofabrication technologies. As this review systematically demonstrates, these 3D microphysiological systems offer unprecedented opportunities to model OA pathogenesis, screen therapeutic compounds, and develop regenerative strategies, owing to their unique capacity to recapitulate native tissue architecture and enable multi-tissue crosstalk.

Advances in osteochondral organoid construction include optimized cell sourcing with MSCs and iPSCs for enhanced differentiation and scalability, sophisticated biomaterial systems ranging from natural hydrogels to synthetic matrices mimicking native ECM, and innovative biofabrication techniques, such as 3D bioprinting and microfluidic platforms, enabling precise spatial organization and vascular integration. These technological synergies have yielded organoid models with improved physiological relevance for studying OA mechanisms, particularly modeling the complex interplay between cartilage degradation, subchondral bone remodeling, and synovial inflammation. The applications of osteochondral organoids span pathomimetic disease modeling and high-throughput drug screening platforms. Notably, gene-edited organoids have provided novel insights into OA-associated genetic variants and mechano-inflammatory pathways, while patient-derived systems show promise for personalized therapeutic testing. In regenerative medicine, transplantation studies reveal the potential of organoids to repair osteochondral defects through biomimetic tissue integration, although long-term functional outcomes require further validation.

Despite these advances, challenges remain in achieving scalable, standardized production with consistent quality, overcoming vascularization and immunomodulatory barriers to clinical translation, and establishing regulatory frameworks for osteochondral organoids. Replicating the complete joint microenvironment, including neural and immune components, also necessitates innovative solutions.

Future progress depends on interdisciplinary integration across several fronts: intelligent systems combining smart materials with organ-on-chip platforms for dynamic microenvironment control; multi-omics and AI-driven approaches to create predictive OA digital twins; and translational initiatives to establish GMP-grade organoid biobanks and clinical validation pathways. As these technologies mature, osteochondral organoids are poised to bridge critical gaps between bench research and clinical practice, enabling precision medicine approaches for OA diagnosis and treatment. This evolving paradigm shifts OA research from observational biology to mechanistic intervention, offering an integrated platform that connects molecular discovery with therapeutic development. Achieving this potential requires continued innovation to overcome technical and translational barriers while addressing ethical considerations in cellular therapeutics.

## References

[B1] Abe K. Yamashita A. Morioka M. Horike N. Takei Y. Koyamatsu S. (2023). Engraftment of allogeneic iPS cell-derived cartilage organoid in a primate model of articular cartilage defect. Nat. Commun. 14, 804. 10.1038/s41467-023-36408-0 36808132 PMC9941131

[B2] Abraham D. M. Herman C. Witek L. Cronstein B. N. Flores R. L. Coelho P. G. (2022). Self-assembling human skeletal organoids for disease modeling and drug testing. J. Biomed. Mat. Res. B Appl. Biomater. 110, 871–884. 10.1002/jbm.b.34968 PMC8854332 34837719

[B3] Abraham N. Kolipaka T. Pandey G. Negi M. Srinivasarao D. A. Srivastava S. (2024). Revolutionizing pancreatic islet organoid transplants: improving engraftment and exploring future frontiers. Life Sci. 343, 122545. 10.1016/j.lfs.2024.122545 38458556

[B4] Alahdal M. Huang R. Duan L. Zhiqin D. Hongwei O. Li W. (2021). Indoleamine 2, 3 dioxygenase 1 impairs chondrogenic differentiation of mesenchymal stem cells in the joint of osteoarthritis mice model. Front. Immunol. 12, 781185. 10.3389/fimmu.2021.781185 34956209 PMC8693178

[B5] Ali E. A. M. Smaida R. Meyer M. Ou W. Li Z. Han Z. (2024). iPSCs chondrogenic differentiation for personalized regenerative medicine: a literature review. Stem Cell. Res. Ther. 15, 185. 10.1186/s13287-024-03794-1 38926793 PMC11210138

[B6] Alnasser S. M. (2025). From gut to liver: organoids as platforms for next-generation toxicology assessment vehicles for xenobiotics. Stem Cell. Res. Ther. 16, 150. 10.1186/s13287-025-04264-y 40140938 PMC11948905

[B7] An X. Wang J. Xu K. Zhao R. C. Su J. (2024). Perspectives on osteoarthritis treatment with mesenchymal stem cells and Radix achyranthis bidentatae. Aging Dis. 15, 1029–1045. 10.14336/AD.2023.0817 37728585 PMC11081162

[B8] An X. Zhou Q. Sheng S. Deng A. Liu H. Wang X. (2025). Enhanced chondrogenic potential and osteoarthritis treatment using cyaonoside A-Induced MSC delivered via a hyaluronic acid-based hydrogel system. Aging Dis. 10.14336/AD.2024.10016 PMC12727091 39908269

[B9] Annan C. Wanying W. Zhengyi M. Yunhu H. Shiting C. Guo L. (2024). Multimaterial 3D and 4D bioprinting of heterogenous constructs for tissue engineering. Adv. Mat. Deerf. Beach Fla 36, e2307686. 10.1002/adma.202307686 37737521

[B10] Banerjee D. Singh Y. P. Datta P. Ozbolat V. O’Donnell A. Yeo M. (2022). Strategies for 3D bioprinting of spheroids: a comprehensive review. Biomaterials 291, 121881. 10.1016/j.biomaterials.2022.121881 36335718

[B11] Bhatia S. N. Ingber D. E. (2014). Microfluidic organs-on-chips. Nat. Biotechnol. 32, 760–772. 10.1038/nbt.2989 25093883

[B12] Bloks N. G. Harissa Z. Mazzini G. Adkar S. S. Dicks A. R. Hajmousa G. (2024). A damaging COL6A3 variant alters the MIR31HG-Regulated response of chondrocytes in neocartilage organoids to hyperphysiologic mechanical loading. Adv. Sci. Weinh. Baden-Wurtt. Ger. 11, e2400720. 10.1002/advs.202400720 PMC11423154 39021299

[B13] Boone I. Houtman E. Tuerlings M. van den Berg J. J. Lehmann J. de Keizer P. L. J. (2025). Development of reliable and high-throughput human biomimetic cartilage and bone models to explore senescence and personalized osteoarthritis treatment options. J. Orthop. Res. Off. Publ. Orthop. Res. Soc. 43, 912–921. 10.1002/jor.26052 PMC11982592 39960283

[B14] Cadena M. A. Sing A. Taylor K. Jin L. Ning L. Salar Amoli M. (2024). A 3D bioprinted cortical organoid platform for modeling human brain development. Adv. Healthc. Mat. 13, e2401603. 10.1002/adhm.202401603 PMC11518656 38815975

[B15] Camarero-Espinosa S. Beeren I. Liu H. Gomes D. B. Zonderland J. Lourenço A. F. H. (2024). 3D niche-inspired scaffolds as a stem cell delivery system for the regeneration of the osteochondral interface. Adv. Mat. Deerf. Beach Fla 36, e2310258. 10.1002/adma.202310258 PMC7619216 38226666

[B16] Chen S. Chen X. Geng Z. Su J. (2022). The horizon of bone organoid: a perspective on construction and application. Bioact. Mat. 18, 15–25. 10.1016/j.bioactmat.2022.01.048 PMC8961298 35387160

[B17] Chen W. Sheng S. Tan K. Wang S. Wu X. Yang J. (2024a). Injectable hydrogels for bone regeneration with tunable degradability via peptide chirality modification. Mat. Horiz. 11, 4367–4377. 10.1039/d4mh00398e 38932613

[B18] Chen X. Zheng J. Yin L. Li Y. Liu H. (2024b). Transplantation of three mesenchymal stem cells for knee osteoarthritis, which cell and type are more beneficial? A systematic review and network meta-analysis. J. Orthop. Surg. 19, 366. 10.1186/s13018-024-04846-1 PMC11188250 38902778

[B19] Chen Y.-C. Gad S. F. Chobisa D. Li Y. Yeo Y. (2021). Local drug delivery systems for inflammatory diseases: status quo, challenges, and opportunities. J. Control. Release Off. J. Control. Release Soc. 330, 438–460. 10.1016/j.jconrel.2020.12.025 33352244

[B20] Chen Z. Bo Q. Wang C. Xu Y. Fei X. Chen R. (2025). Single BMSC-Derived cartilage organoids for gradient heterogeneous osteochondral regeneration by leveraging native vascular microenvironment. J. Nanobiotechnology 23, 325. 10.1186/s12951-025-03403-0 40301867 PMC12042616

[B21] Chijimatsu R. Ikeya M. Yasui Y. Ikeda Y. Ebina K. Moriguchi Y. (2017). Characterization of mesenchymal stem cell-like cells derived from human iPSCs via neural crest development and their application for osteochondral repair. Stem Cells Int. 2017, 1–18. 10.1155/2017/1960965 PMC5451770 28607560

[B22] Conceição F. Meneses J. Lebre F. Becker M. Araújo-Gomes N. Vos R. (2025). Sex-stratified osteochondral organ-on-chip model reveals sex-specific responses to inflammatory stimulation. Mat. Today Bio 32, 101728. 10.1016/j.mtbio.2025.101728 PMC12000750 40242482

[B23] Corrado F. Di Maio L. Palmero P. Coppola B. Abbas Z. La Gatta A. (2025). Vat photo-polymerization 3D printing of gradient scaffolds for osteochondral tissue regeneration. Acta Biomater. 200, 67–86. 10.1016/j.actbio.2025.05.042 40414264

[B24] Davoodi E. Sarikhani E. Montazerian H. Ahadian S. Costantini M. Swieszkowski W. (2020). Extrusion and microfluidic-based bioprinting to fabricate biomimetic tissues and organs. Adv. Mat. Technol. 5, 1901044. 10.1002/admt.201901044 PMC7567134 33072855

[B25] Dehghani Nazhvani F. Mohammadi Amirabad L. Azari A. Namazi H. Hosseinzadeh S. Samanipour R. (2021). Effects of *in vitro* low oxygen tension preconditioning of buccal fat pad stem cells on *in vivo* articular cartilage tissue repair. Life Sci. 280, 119728. 10.1016/j.lfs.2021.119728 34144057

[B26] Ding Z. Yan Z. Yuan X. Tian G. Wu J. Fu L. (2024). Apoptotic extracellular vesicles derived from hypoxia-preconditioned mesenchymal stem cells within a modified gelatine hydrogel promote osteochondral regeneration by enhancing stem cell activity and regulating immunity. J. Nanobiotechnology 22, 74. 10.1186/s12951-024-02333-7 38395929 PMC10885680

[B27] Dönges L. Damle A. Mainardi A. Bock T. Schönenberger M. Martin I. (2024). Engineered human osteoarthritic cartilage organoids. Biomaterials 308, 122549. 10.1016/j.biomaterials.2024.122549 38554643

[B28] Dou D. Lu J. Dou J. Huo Y. Gong X. Zhang X. (2025). Global regulatory considerations and practices for tumorigenicity evaluation of cell-based therapy. Regul. Toxicol. Pharmacol. RTP 156, 105769. 10.1016/j.yrtph.2024.105769 39743127

[B29] Elder S. Chenault H. Gloth P. Webb K. Recinos R. Wright E. (2018). Effects of antigen removal on a porcine osteochondral xenograft for articular cartilage repair. J. Biomed. Mat. Res. A 106, 2251–2260. 10.1002/jbm.a.36411 PMC6779129 29577591

[B30] El-Tanani M. Satyam S. M. Rabbani S. A. El-Tanani Y. Aljabali A. A. A. Al Faouri I. (2025). Revolutionizing drug delivery: the impact of advanced materials science and technology on precision medicine. Pharmaceutics 17, 375. 10.3390/pharmaceutics17030375 40143038 PMC11944361

[B31] Fatehullah A. Tan S. H. Barker N. (2016). Organoids as an *in vitro* model of human development and disease. Nat. Cell. Biol. 18, 246–254. 10.1038/ncb3312 26911908

[B32] Franchi F. Schneider D. J. Prats J. Fan W. Rollini F. Been L. (2022). Pharmacokinetic and pharmacodynamic profiles of a novel phospholipid-aspirin complex liquid formulation and low dose enteric-coated aspirin: results from a prospective, randomized, crossover study. J. Thromb. Thrombolysis 54, 373–381. 10.1007/s11239-022-02687-5 36036856 PMC9421621

[B33] Gao Q. Wang J. Zhang H. Wang J. Jing Y. Su J. (2025). Organoid vascularization: strategies and applications. Adv. Healthc. Mat., e2500301. 10.1002/adhm.202500301 40285576

[B34] Gaykema L. H. van Nieuwland R. Y. Lievers E. Moerkerk W. B. J. de Klerk J. A. Dumas S. J. (2024). T-Cell mediated immune rejection of Beta-2-Microglobulin knockout induced pluripotent stem cell-derived kidney organoids. Stem Cells Transl. Med. 13, 69–82. 10.1093/stcltm/szad069 37843402 PMC10785221

[B35] González-Guede I. Garriguez-Perez D. Fernandez-Gutierrez B. (2024). Osteochondral Tissue-On-a-Chip: a novel model for osteoarthritis research. Int. J. Mol. Sci. 25, 9834. 10.3390/ijms25189834 39337321 PMC11432185

[B36] Gonzalez-Rubio J. Zeevaert K. Buhl E. M. Schedel M. Jockenhoevel S. Cornelissen C. G. (2025). iPSC-derived mesenchymal stromal cells stimulate neovascularization less than their primary counterparts. Life Sci. 361, 123298. 10.1016/j.lfs.2024.123298 39647809

[B37] Grässel S. Zaucke F. Madry H. (2021). Osteoarthritis: novel molecular mechanisms increase our understanding of the disease pathology. J. Clin. Med. 10, 1938. 10.3390/jcm10091938 33946429 PMC8125020

[B38] Grottkau B. E. Hui Z. Pang Y. (2022). Articular cartilage regeneration through bioassembling spherical micro-cartilage building blocks. Cells 11, 3244. 10.3390/cells11203244 36291114 PMC9600996

[B39] Gu Y. Zhang W. Wu X. Zhang Y. Xu K. Su J. (2023). Organoid assessment technologies. Clin. Transl. Med. 13, e1499. 10.1002/ctm2.1499 38115706 PMC10731122

[B40] Haack A. J. Brown L. G. Goldstein A. J. Mulimani P. Berthier J. Viswanathan A. R. (2025). Suspended tissue open microfluidic patterning (STOMP). Adv. Sci. Weinh. Baden-Wurtt. Ger. 12, e2501148. 10.1002/advs.202501148 PMC12225008 40298902

[B41] Hall G. N. Tam W. L. Andrikopoulos K. S. Casas-Fraile L. Voyiatzis G. A. Geris L. (2021). Patterned, organoid-based cartilaginous implants exhibit zone specific functionality forming osteochondral-like tissues *in vivo* . Biomaterials 273, 120820. 10.1016/j.biomaterials.2021.120820 33872857

[B42] Hamilton M. Wang J. Dhar P. Stehno-Bittel L. (2023). Controlled-release hydrogel microspheres to deliver multipotent stem cells for treatment of knee osteoarthritis. Bioeng. Basel Switz. 10, 1315. 10.3390/bioengineering10111315 PMC10669156 38002439

[B43] He S. Liang W. Tang Y. Zhang J. Wang R. Quan L. (2025). Robust super-structured porous hydrogel enables bioadaptive repair of dynamic soft tissue. Nat. Commun. 16, 3198. 10.1038/s41467-025-58062-4 40180956 PMC11968947

[B44] He Y. Li H. Yu Z. Li L. Chen X. Yang A. (2022). Exosomal let-7f-5p derived from mineralized osteoblasts promotes the angiogenesis of endothelial cells *via* the DUSP1/Erk1/2 signaling pathway. J. Tissue Eng. Regen. Med. 16, 1184–1195. 10.1002/term.3358 36348261

[B45] Hill M. Andrews-Pfannkoch C. Atherton E. Knudsen T. Trncic E. Marmorstein A. D. (2024). Detection of residual iPSCs following differentiation of iPSC-Derived retinal pigment epithelial cells. J. Ocul. Pharmacol. Ther. Off. J. Assoc. Ocul. Pharmacol. Ther. 40, 680–687. 10.1089/jop.2024.0130 PMC11698679 39358867

[B46] Horvath P. Aulner N. Bickle M. Davies A. M. Nery E. D. Ebner D. (2016). Screening out irrelevant cell-based models of disease. Nat. Rev. Drug Discov. 15, 751–769. 10.1038/nrd.2016.175 27616293

[B47] Hu Y. Zhu T. Cui H. Cui H. (2025). Integrating 3D bioprinting and organoids to better recapitulate the complexity of cellular microenvironments for tissue engineering. Adv. Healthc. Mat. 14, e2403762. 10.1002/adhm.202403762 39648636

[B48] Huang S. Gao D. Li Z. He H. Yu X. You X. (2025). Neuronal guidance factor Sema3A inhibits neurite ingrowth and prevents chondrocyte hypertrophy in the degeneration of knee cartilage in mice, monkeys and humans. Bone Res. 13, 4. 10.1038/s41413-024-00382-0 39746903 PMC11695747

[B49] Huang Y. Huang Z. Tang Z. Chen Y. Huang M. Liu H. (2021). Research progress, challenges, and breakthroughs of organoids as disease models. Front. Cell. Dev. Biol. 9, 740574. 10.3389/fcell.2021.740574 34869324 PMC8635113

[B50] Hunter D. J. March L. Chew M. (2020). Osteoarthritis in 2020 and beyond: a lancet commission. Lancet lond. Engl. 396, 1711–1712. 10.1016/S0140-6736(20)32230-3 33159851

[B51] Jia L. Zhang P. Ci Z. Hao X. Bai B. Zhang W. (2022). Acellular cartilage matrix biomimetic scaffold with immediate enrichment of autologous bone marrow mononuclear cells to repair articular cartilage defects. Mat. Today Bio 15, 100310. 10.1016/j.mtbio.2022.100310 PMC9168693 35677810

[B52] Kaur S. Kaur I. Rawal P. Tripathi D. M. Vasudevan A. (2021). Non-matrigel scaffolds for organoid cultures. Cancer Lett. 504, 58–66. 10.1016/j.canlet.2021.01.025 33582211

[B53] Kesharwani A. Tani S. Nishikawa M. Sakai Y. Okada H. Ohba S. (2025). Modeling vascular dynamics at the initial stage of endochondral ossification on a microfluidic chip using a human embryonic-stem-cell-derived organoid. Regen. Ther. 28, 90–100. 10.1016/j.reth.2024.11.018 39703814 PMC11655692

[B54] Kim H.-S. Li C. J. Park S.-M. Kim K. W. Mo J.-H. Jin G.-Z. (2024a). Development of an injectable biphasic hyaluronic acid-based hydrogel with stress relaxation properties for cartilage regeneration. Adv. Healthc. Mat. 13, e2400043. 10.1002/adhm.202400043 38569577

[B55] Kim J. Kim J.-S. Kim D. Bello A. B. Kim B. J. Cha B.-H. (2024b). Therapeutic potential of mesenchymal stem cells from human iPSC-derived teratomas for osteochondral defect regeneration. Bioeng. Transl. Med. 9, e10629. 10.1002/btm2.10629 38435815 PMC10905541

[B56] Kronemberger G. S. Spagnuolo F. D. Karam A. S. Chattahy K. Storey K. J. Kelly D. J. (2025). Growth factor stimulation regimes to support the development and fusion of cartilage microtissues. Tissue Eng. Part C Methods 31, 36–48. 10.1089/ten.tec.2024.0309 39813639

[B57] Lai J. Liu Y. Lu G. Yung P. Wang X. Tuan R. S. (2024). 4D bioprinting of programmed dynamic tissues. Bioact. Mat. 37, 348–377. 10.1016/j.bioactmat.2024.03.033 PMC11061618 38694766

[B58] Lee J. Lee E. Huh S. J. Kang J. I. Park K. M. Byun H. (2024). Composite spheroid-laden bilayer hydrogel for engineering three-dimensional osteochondral tissue. Tissue Eng. Part A 30, 225–243. 10.1089/ten.TEA.2023.0299 38062771

[B59] Li M. Hu X. Liu X. Zhao L. Zhao W. Li Y. (2025a). 3D bioprinted piezoelectric hydrogel synergized with LIPUS to promote bone regeneration. Mat. Today Bio 31, 101604. 10.1016/j.mtbio.2025.101604 PMC11891151 40066077

[B60] Li Y. Huang D. Zhang Y. Xiao Y. Zhang X. (2025b). Microfluidic-assisted engineering of hydrogels with microscale complexity. Acta Biomater. 199, 1–17. 10.1016/j.actbio.2025.05.023 40349902

[B61] Limraksasin P. Kondo T. Zhang M. Okawa H. Osathanon T. Pavasant P. (2020). *In vitro* fabrication of hybrid bone/cartilage complex using mouse induced pluripotent stem cells. Int. J. Mol. Sci. 21, 581. 10.3390/ijms21020581 31963264 PMC7014254

[B62] Lin H. Cheng J. Zhu C. Yang Z. Shen Q. Zou Y. (2025). Artificial intelligence-enabled quantitative assessment and intervention for heart inflammation model organoids. Angew. Chem. Int. Ed. Engl. 64, e202503252. 10.1002/anie.202503252 40208199

[B63] Lin Z. Li Z. Li E. N. Li X. Del Duke C. J. Shen H. (2019). Osteochondral tissue chip derived from iPSCs: modeling OA pathologies and testing drugs. Front. Bioeng. Biotechnol. 7, 411. 10.3389/fbioe.2019.00411 31921815 PMC6930794

[B64] Liu C. Yu Q. Yuan Z. Guo Q. Liao X. Han F. (2023). Engineering the viscoelasticity of gelatin methacryloyl (GelMA) hydrogels *via* small “dynamic bridges” to regulate BMSC behaviors for osteochondral regeneration. Bioact. Mat. 25, 445–459. 10.1016/j.bioactmat.2022.07.031 PMC10087107 37056254

[B65] Liu D. Wang X. Gao C. Zhang Z. Wang Q. Pei Y. (2024a). Biodegradable piezoelectric-conductive integrated hydrogel scaffold for repair of osteochondral defects. Adv. Mat. Deerf. Beach Fla 36, e2409400. 10.1002/adma.202409400 39267457

[B66] Liu H. Gan Z. Qin X. Wang Y. Qin J. (2024b). Advances in microfluidic technologies in organoid research. Adv. Healthc. Mat. 13, e2302686. 10.1002/adhm.202302686 38134345

[B67] Liu H. Wu X. Liu R. Wang W. Zhang D. Jiang Q. (2024c). Cartilage-on-a-chip with magneto-mechanical transformation for osteoarthritis recruitment. Bioact. Mat. 33, 61–68. 10.1016/j.bioactmat.2023.10.030 PMC10661690 38024232

[B68] Liu X. Zheng C. Luo X. Wang X. Jiang H. (2019). Recent advances of collagen-based biomaterials: multi-Hierarchical structure, modification and biomedical applications. Mat. Sci. Eng. C Mat. Biol. Appl. 99, 1509–1522. 10.1016/j.msec.2019.02.070 30889687

[B69] Liuyang S. Wang G. Wang Y. He H. Lyu Y. Cheng L. (2023). Highly efficient and rapid generation of human pluripotent stem cells by chemical reprogramming. Cell. Stem Cell. 30, 450–459.e9. 10.1016/j.stem.2023.02.008 36944335

[B70] Lopa S. Mondadori C. Mainardi V. L. Talò G. Costantini M. Candrian C. (2018). Translational application of microfluidics and bioprinting for stem cell-based cartilage repair. Stem Cells Int. 2018, 6594841–14. 10.1155/2018/6594841 29535776 PMC5838503

[B71] Luo Q. Shang K. Zhu J. Wu Z. Cao T. Ahmed A. A. Q. (2023). Biomimetic cell culture for cell adhesive propagation for tissue engineering strategies. Mat. Horiz. 10, 4662–4685. 10.1039/d3mh00849e 37705440

[B72] Ma M. Zou F. Abudureheman B. Han F. Xu G. Xie Y. (2023). Magnetic microcarriers with accurate localization and proliferation of mesenchymal stem cell for cartilage defects repairing. ACS Nano 17, 6373–6386. 10.1021/acsnano.2c10995 36961738

[B73] Maherani M. Eslami H. Poursamar S. A. Ansari M. (2024). A modular approach to 3D-printed bilayer composite scaffolds for osteochondral tissue engineering. J. Mat. Sci. Mat. Med. 35, 62. 10.1007/s10856-024-06824-9 PMC11456551 39370474

[B74] Mainardi A. Börsch A. Occhetta P. Ivanek R. Ehrbar M. Krattiger L. (2025). An organ-on-chip platform for strain-controlled, tissue-specific compression of cartilage and mineralized osteochondral interface to study mechanical overloading in osteoarthritis. Adv. Healthc. Mat., e2501588. 10.1002/adhm.202501588 PMC12417778 40556597

[B75] Maramraju S. Kowalczewski A. Kaza A. Liu X. Singaraju J. P. Albert M. V. (2024). AI-organoid integrated systems for biomedical studies and applications. Bioeng. Transl. Med. 9, e10641. 10.1002/btm2.10641 38435826 PMC10905559

[B76] Matai I. Kaur G. Seyedsalehi A. McClinton A. Laurencin C. T. (2020). Progress in 3D bioprinting technology for tissue/organ regenerative engineering. Biomaterials 226, 119536. 10.1016/j.biomaterials.2019.119536 31648135

[B77] Mirazi H. Wood S. T. (2025). Microfluidic chip-based co-culture system for modeling human joint inflammation in osteoarthritis research. Front. Pharmacol. 16, 1579228. 10.3389/fphar.2025.1579228 40271077 PMC12015981

[B78] Mo F. Jiang K. Zhao D. Wang Y. Song J. Tan W. (2021). DNA hydrogel-based gene editing and drug delivery systems. Adv. Drug Deliv. Rev. 168, 79–98. 10.1016/j.addr.2020.07.018 32712197

[B79] Mondadori C. Palombella S. Salehi S. Talò G. Visone R. Rasponi M. (2021). Recapitulating monocyte extravasation to the synovium in an organotypic microfluidic model of the articular joint. Biofabrication 13, 045001. 10.1088/1758-5090/ac0c5e 34139683

[B80] Negishi Y. Adili A. de Vega S. Momoeda M. Kaneko H. Cilek M. Z. (2024). IL-6 reduces spheroid sizes of osteophytic cells derived from osteoarthritis knee joint *via* induction of apoptosis. Am. J. Pathol. 194, 135–149. 10.1016/j.ajpath.2023.10.005 37918800

[B81] Ni T. Liu M. Zhang Y. Cao Y. Pei R. (2020). 3D bioprinting of bone marrow mesenchymal stem cell-laden silk fibroin double network scaffolds for cartilage tissue repair. Bioconjug. Chem. 31, 1938–1947. 10.1021/acs.bioconjchem.0c00298 32644779

[B82] Noh S. Jin Y. J. Shin D. I. Kwon H. J. Yun H.-W. Kim K. M. (2023). Selective extracellular matrix guided mesenchymal stem cell self-aggregate engineering for replication of meniscal zonal tissue gradient in a porcine meniscectomy model. Adv. Healthc. Mat. 12, e2301180. 10.1002/adhm.202301180 37463568

[B83] Notoh H. Yamasaki S. Suzuki N. Suzuki A. Okamoto S. Kanematsu T. (2024). Basement membrane extract potentiates the endochondral ossification phenotype of bone marrow-derived mesenchymal stem cell-based cartilage organoids. Biochem. Biophys. Res. Commun. 701, 149583. 10.1016/j.bbrc.2024.149583 38330731

[B84] O’Connor S. K. Katz D. B. Oswald S. J. Groneck L. Guilak F. (2021). Formation of osteochondral organoids from murine induced pluripotent stem cells. Tissue Eng. Part A 27, 1099–1109. 10.1089/ten.TEA.2020.0273 33191853 PMC8392116

[B85] O’Donnell B. T. Al-Ghadban S. Ives C. J. L’Ecuyer M. P. Monjure T. A. Romero-Lopez M. (2020). Adipose tissue-derived stem cells retain their adipocyte differentiation potential in three-dimensional hydrogels and bioreactors. Biomolecules 10, 1070. 10.3390/biom10071070 32709032 PMC7408056

[B86] Patel S. N. Ishahak M. Chaimov D. Velraj A. LaShoto D. Hagan D. W. (2021). Organoid microphysiological system preserves pancreatic islet function within 3D matrix. Sci. Adv. 7, eaba5515. 10.1126/sciadv.aba5515 33579705 PMC7880596

[B87] Petta D. D’Arrigo D. Salehi S. Talò G. Bonetti L. Vanoni M. (2024). A personalized osteoarthritic joint-on-a-chip as a screening platform for biological treatments. Mat. Today Bio 26, 101072. 10.1016/j.mtbio.2024.101072 PMC11097088 38757057

[B88] Piraino F. Costa M. Meyer M. Cornish G. Ceroni C. Garnier V. (2024). Organoid models: the future companions of personalized drug development. Biofabrication 16, 032009. 10.1088/1758-5090/ad3e30 38608454

[B89] Qiao L. Zhao Y. Zhang M. Tao Y. Xiao Y. Zhang N. (2024). Preparation strategies, functional regulation, and applications of multifunctional nanomaterials-based DNA hydrogels. Small Methods 8, e2301261. 10.1002/smtd.202301261 38010956

[B90] Quintard C. Tubbs E. Jonsson G. Jiao J. Wang J. Werschler N. (2024). A microfluidic platform integrating functional vascularized organoids-on-chip. Nat. Commun. 15, 1452. 10.1038/s41467-024-45710-4 38365780 PMC10873332

[B91] Rodríguez Ruiz A. van Hoolwerff M. Sprangers S. Suchiman E. Schoenmaker T. Dibbets-Schneider P. (2022). Mutation in the CCAL1 locus accounts for bidirectional process of human subchondral bone turnover and cartilage mineralization. Rheumatol. Oxf. Engl. 62, 360–372. 10.1093/rheumatology/keac232 PMC9788812 35412619

[B92] Rong M. Liu D. Xu X. Li A. Bai Y. Yang G. (2024). A superparamagnetic composite hydrogel scaffold as *in vivo* dynamic monitorable Theranostic platform for osteoarthritis regeneration. Adv. Mat. Deerf. Beach Fla 36, e2405641. 10.1002/adma.202405641 38877353

[B93] Schwab A. Wesdorp M. A. Xu J. Abinzano F. Loebel C. Falandt M. (2023). Modulating design parameters to drive cell invasion into hydrogels for osteochondral tissue formation. J. Orthop. Transl. 41, 42–53. 10.1016/j.jot.2023.07.001 PMC10485598 37691639

[B94] Shen C. Wang J. Li G. Hao S. Wu Y. Song P. (2024). Boosting cartilage repair with silk fibroin-DNA hydrogel-based cartilage organoid precursor. Bioact. Mat. 35, 429–444. 10.1016/j.bioactmat.2024.02.016 PMC10881360 38390528

[B95] Shen C. Zhou Z. Li R. Yang S. Zhou D. Zhou F. (2025). Silk fibroin-based hydrogels for cartilage organoids in osteoarthritis treatment. Theranostics 15, 560–584. 10.7150/thno.103491 39744693 PMC11671376

[B96] Shi X. Zhang K. Yu F. Qi Q. Cai X. Zhang Y. (2024). Advancements and innovative strategies in induced pluripotent stem cell-derived mesenchymal stem cell therapy: a comprehensive review. Stem Cells Int. 2024, 4073485. 10.1155/2024/4073485 39377039 PMC11458320

[B97] Skoracka J. Bajewska K. Kulawik M. Suchorska W. Kulcenty K. (2024). Advances in cartilage tissue regeneration: a review of stem cell therapies, tissue engineering, biomaterials, and clinical trials. EXCLI J. 23, 1170–1182. 10.17179/excli2024-7088 39391058 PMC11464958

[B98] Smadja D. M. Berkane Y. Bentounes N. K. Rancic J. Cras A. Pinault C. (2025). Immune-privileged cord blood-derived endothelial colony-forming cells: advancing immunomodulation and vascular regeneration. Angiogenesis 28, 19. 10.1007/s10456-025-09973-9 40047974 PMC11885380

[B99] Smith K. W. Y. Fung S. L. Wu H.-F. Chiesa I. Vozzi G. De Maria C. (2025). Developing an *in vitro* osteochondral micro-physiological system for modeling cartilage-bone crosstalk in arthritis. Front. Immunol. 16, 1495613. 10.3389/fimmu.2025.1495613 40491903 PMC12146386

[B100] Su X. Wang T. Guo S. (2021). Applications of 3D printed bone tissue engineering scaffolds in the stem cell field. Regen. Ther. 16, 63–72. 10.1016/j.reth.2021.01.007 33598507 PMC7868584

[B101] Sun N. Wang C. Edwards W. Wang Y. Lu X. L. Gu C. (2025). Nanoneedle-based electroporation for efficient manufacturing of human primary chimeric antigen receptor regulatory T-Cells. Adv. Sci. Weinh. Baden-Wurtt. Ger. 12, e2416066. 10.1002/advs.202416066 PMC12140303 40231643

[B102] Taheem D. K. Jell G. Gentleman E. (2020). Hypoxia inducible Factor-1α in osteochondral tissue engineering. Tissue Eng. Part B Rev. 26, 105–115. 10.1089/ten.TEB.2019.0283 31774026 PMC7166133

[B103] Tam W. L. Freitas Mendes L. Chen X. Lesage R. Van Hoven I. Leysen E. (2021). Human pluripotent stem cell-derived cartilaginous organoids promote scaffold-free healing of critical size long bone defects. Stem Cell. Res. Ther. 12, 513. 10.1186/s13287-021-02580-7 34563248 PMC8466996

[B104] Thompson C. L. Hopkins T. Bevan C. Screen H. R. C. Wright K. T. Knight M. M. (2023). Human vascularised synovium-on-a-chip: a mechanically stimulated, microfluidic model to investigate synovial inflammation and monocyte recruitment. Biomed. Mat. Bristol Engl. 18, 065013. 10.1088/1748-605X/acf976 37703884

[B105] Tolabi H. Davari N. Khajehmohammadi M. Malektaj H. Nazemi K. Vahedi S. (2023). Progress of microfluidic hydrogel-based scaffolds and organ-on-chips for the cartilage tissue engineering. Adv. Mat. Deerf. Beach Fla 35, e2208852. 10.1002/adma.202208852 36633376

[B106] Tong X. Ayushman M. Lee H.-P. Yang F. (2025). Tuning local matrix compliance accelerates mesenchymal stem cell chondrogenesis in 3D sliding hydrogels. Biomaterials 317, 123112. 10.1016/j.biomaterials.2025.123112 39827509 PMC12740556

[B107] Upadhyay U. Kolla S. Maredupaka S. Priya S. Srinivasulu K. Chelluri L. K. (2024). Development of an alginate-chitosan biopolymer composite with dECM bioink additive for organ-on-a-chip articular cartilage. Sci. Rep. 14, 11765. 10.1038/s41598-024-62656-1 38782958 PMC11116456

[B108] Urlić I. Ivković A. (2021). Cell sources for cartilage repair-biological and clinical perspective. Cells 10, 2496. 10.3390/cells10092496 34572145 PMC8468484

[B109] van Hoolwerff M. Rodríguez Ruiz A. Bouma M. Suchiman H. E. D. Koning R. I. Jost C. R. (2021). High-impact FN1 mutation decreases chondrogenic potential and affects cartilage deposition *via* decreased binding to collagen type II. Sci. Adv. 7, eabg8583. 10.1126/sciadv.abg8583 34739320 PMC8570604

[B110] Vanlauwe F. Dermaux C. Shamieva S. Vermeiren S. Van Vlierberghe S. Blondeel P. (2024). Small molecular weight alginate gel porogen for the 3D bioprinting of microvasculature. Front. Bioeng. Biotechnol. 12, 1452477. 10.3389/fbioe.2024.1452477 39380897 PMC11458444

[B111] Wang J. Chen X. Li R. Wang S. Geng Z. Shi Z. (2025a). Standardization and consensus in the development and application of bone organoids. Theranostics 15, 682–706. 10.7150/thno.105840 39744680 PMC11671374

[B112] Wang J. Wu Y. Li G. Zhou F. Wu X. Wang M. (2024a). Engineering large-scale self-mineralizing bone organoids with bone matrix-inspired hydroxyapatite hybrid bioinks. Adv. Mat. Deerf. Beach Fla 36, e2309875. 10.1002/adma.202309875 38642033

[B113] Wang T. Zhang M. Guo J. Wei H. Li W. Luo Y. (2025b). Alginate/Bacterial cellulose/GelMA scaffolds with aligned nanopatterns and hollow channel networks for vascularized bone repair. Int. J. Biol. Macromol. 308, 142578. 10.1016/j.ijbiomac.2025.142578 40154692

[B114] Wang W. Liang X. Zheng K. Ge G. Chen X. Xu Y. (2022). Horizon of exosome-mediated bone tissue regeneration: the all-rounder role in biomaterial engineering. Mat. Today Bio 16, 100355. 10.1016/j.mtbio.2022.100355 PMC9304878 35875196

[B115] Wang W. Liu Z. Zhu J. Zhen H. Qi M. Luo J. (2024b). Macrophage tracking with USPIO imaging and T2 mapping predicts immune rejection of transplanted stem cells. Sci. Rep. 14, 29162. 10.1038/s41598-024-80750-2 39587241 PMC11589617

[B116] Wang X. Zeng Z. Li D. Wang K. Zhang W. Yu Y. (2025c). Advancements and challenges in immune protection strategies for islet transplantation. J. Diabetes 17, e70048. 10.1111/1753-0407.70048 39829227 PMC11744047

[B117] Wei W. Liu W. Kang H. Zhang X. Yu R. Liu J. (2023). A one-stone-two-birds strategy for osteochondral regeneration based on a 3D printable biomimetic scaffold with kartogenin biochemical stimuli gradient. Adv. Healthc. Mat. 12, e2300108. 10.1002/adhm.202300108 36763493

[B118] Wu J. Y. Yeager K. Tavakol D. N. Morsink M. Wang B. Soni R. K. (2023). Directed differentiation of human iPSCs into mesenchymal lineages by optogenetic control of TGF-β signaling. Cell. Rep. 42, 112509. 10.1016/j.celrep.2023.112509 37178118 PMC10278972

[B119] Xiao Y. Ma J. Yuan X. Wang H. Ma F. Wu J. (2025). Acid-triggered dual-functional hydrogel platform for enhanced bone regeneration. Adv. Sci. Weinh. Baden-Wurtt. Ger. 12, e2415772. 10.1002/advs.202415772 PMC11923904 39868910

[B120] Xu R. Ooi H. S. Bian L. Ouyang L. Sun W. (2025). Dynamic hydrogels for biofabrication: a review. Biomaterials 320, 123266. 10.1016/j.biomaterials.2025.123266 40120174

[B121] Yang J. Wang H. Huang W. Peng K. Shi R. Tian W. (2023a). A natural polymer-based hydrogel with shape controllability and high toughness and its application to efficient osteochondral regeneration. Mat. Horiz. 10, 3797–3806. 10.1039/d3mh00544e 37416948

[B122] Yang Q. Li M. Xiao Z. Feng Y. Lei L. Li S. (2025). A new perspective on precision medicine: the power of digital organoids. Biomater. Res. 29, 0171. 10.34133/bmr.0171 40129676 PMC11931648

[B123] Yang Z. Wang B. Liu W. Li X. Liang K. Fan Z. (2023b). *In situ* self-assembled organoid for osteochondral tissue regeneration with dual functional units. Bioact. Mat. 27, 200–215. 10.1016/j.bioactmat.2023.04.002 PMC10121637 37096194

[B124] Yang Z. Wu Y. Neo S. H. Yang D. Jeon H. Tee C. A. (2024). Size-based microfluidic-enriched mesenchymal stem cell subpopulations enhance articular cartilage repair. Am. J. Sports Med. 52, 503–515. 10.1177/03635465231214431 38186352

[B125] Yao H. Wang C. Zhang Y. Wan Y. Min Q. (2023a). Manufacture of bilayered composite hydrogels with strong, elastic, and tough properties for osteochondral repair applications. Biomim. Basel Switz. 8, 203. 10.3390/biomimetics8020203 PMC10204375 37218789

[B126] Yao L. Lu J. Zhong L. Wei Y. Gui T. Wang L. (2023b). Activin A marks a novel progenitor cell population during fracture healing and reveals a therapeutic strategy. eLife 12, e89822. 10.7554/eLife.89822 38079220 PMC10783872

[B127] Yao Q. Cheng S. Pan Q. Yu J. Cao G. Li L. (2024). Organoids: development and applications in disease models, drug discovery, precision medicine, and regenerative medicine. MedComm 5, e735. 10.1002/mco2.735 39309690 PMC11416091

[B128] Yao Q. Wu X. Tao C. Gong W. Chen M. Qu M. (2023c). Osteoarthritis: pathogenic signaling pathways and therapeutic targets. Signal Transduct. Target. Ther. 8, 56. 10.1038/s41392-023-01330-w 36737426 PMC9898571

[B129] Yao Z. Qi W. Zhang H. Zhang Z. Liu L. Shao Y. (2023d). Down-regulated GAS6 impairs synovial macrophage efferocytosis and promotes obesity-associated osteoarthritis. eLife 12, e83069. 10.7554/eLife.83069 37144868 PMC10191622

[B130] Yarali E. Mirzaali M. J. Ghalayaniesfahani A. Accardo A. Diaz-Payno P. J. Zadpoor A. A. (2024). 4D printing for biomedical applications. Adv. Mat. Deerf. Beach Fla 36, e2402301. 10.1002/adma.202402301 38580291

[B131] Yin P. Su W. Li T. Wang L. Pan J. Wu X. (2023). A modular hydrogel bioink containing microsphere-embedded chondrocytes for 3D-printed multiscale composite scaffolds for cartilage repair. iScience 26, 107349. 10.1016/j.isci.2023.107349 37539040 PMC10393809

[B132] Yu S. P.-C. Hunter D. J. (2015). Emerging drugs for the treatment of knee osteoarthritis. Expert Opin. Emerg. Drugs 20, 361–378. 10.1517/14728214.2015.1037275 25865855

[B133] Zeng D. Chen Y. Liao Z. Wei G. Huang X. Liang R. (2023). Cartilage organoids and osteoarthritis research: a narrative review. Front. Bioeng. Biotechnol. 11, 1278692. 10.3389/fbioe.2023.1278692 38026876 PMC10666186

[B134] Zhang J. Griesbach J. Ganeyev M. Zehnder A.-K. Zeng P. Schädli G. N. (2022a). Long-term mechanical loading is required for the formation of 3D bioprinted functional osteocyte bone organoids. Biofabrication 14, 035018. 10.1088/1758-5090/ac73b9 35617929

[B135] Zhang J. Wang L. Song Q. Xiao M. Gao J. Cao X. (2022b). Organoids in recapitulating tumorigenesis driven by risk factors: current trends and future perspectives. Int. J. Biol. Sci. 18, 2729–2743. 10.7150/ijbs.70406 35541903 PMC9066109

[B136] Zhang J.-Y. Xiang X.-N. Yu X. Liu Y. Jiang H.-Y. Peng J.-L. (2024). Mechanisms and applications of the regenerative capacity of platelets-based therapy in knee osteoarthritis. Biomed. Pharmacother. Biomedecine Pharmacother. 178, 117226. 10.1016/j.biopha.2024.117226 39079262

[B137] Zhang L. Dai W. Gao C. Wei W. Huang R. Zhang X. (2023a). Multileveled hierarchical hydrogel with continuous biophysical and biochemical gradients for enhanced repair of full-thickness osteochondral defect. Adv. Mat. Deerf. Beach Fla 35, e2209565. 10.1002/adma.202209565 36870325

[B138] Zhang X. Wu S. Zhu Y. Chu C.-Q. (2021). Exploiting joint-resident stem cells by exogenous SOX9 for cartilage regeneration for therapy of osteoarthritis. Front. Med. 8, 622609. 10.3389/fmed.2021.622609 PMC7928416 33681252

[B139] Zhang Y. Fang Q. Peng Y. Liu H. Tang J. Ma R. (2025). Establishment and characterization of an inflammatory cartilaginous organoids model for organoid transplantation study. J. Orthop. Transl. 52, 376–386. 10.1016/j.jot.2025.05.002 PMC12138948 40476067

[B140] Zhang Y. Zhuang H. Ren X. Jiang F. Zhou P. (2023b). Therapeutic effects of different intervention forms of human umbilical cord mesenchymal stem cells in the treatment of osteoarthritis. Front. Cell. Dev. Biol. 11, 1246504. 10.3389/fcell.2023.1246504 37635870 PMC10448389

[B141] Zhao H. Chen Y. Shao L. Xie M. Nie J. Qiu J. (2018). Airflow-assisted 3D bioprinting of human heterogeneous microspheroidal organoids with microfluidic nozzle. Small Weinh. Bergstr. Ger. 14, e1802630. 10.1002/smll.201802630 30133151

[B142] Zhu D. Trinh P. Liu E. Yang F. (2023). Cell-cell interactions enhance cartilage zonal development in 3D gradient hydrogels. ACS Biomater. Sci. Eng. 9, 831–843. 10.1021/acsbiomaterials.2c00469 36629329

[B143] Zhu M. Zhang H. Zhou Q. Sheng S. Gao Q. Geng Z. (2025). Dynamic GelMA/DNA dual-network hydrogels promote woven bone organoid formation and enhance bone regeneration. Adv. Mat. Deerf. Beach Fla 37, e2501254. 10.1002/adma.202501254 40123197

[B144] Zhu S. Xuan J. Shentu Y. Kida K. Kobayashi M. Wang W. (2024). Effect of chitin-architected spatiotemporal three-dimensional culture microenvironments on human umbilical cord-derived mesenchymal stem cells. Bioact. Mat. 35, 291–305. 10.1016/j.bioactmat.2024.01.014 PMC10869358 38370866

[B145] Zupan J. Stražar K. (2024). Synovium-derived and bone-derived mesenchymal stem/stromal cells from early OA patients show comparable *in vitro* properties to those of Non-OA patients. Cells 13, 1238. 10.3390/cells13151238 39120270 PMC11311703

